# Self-Supported *N*-Heterocyclic Carbenes and Their Use as Organocatalysts

**DOI:** 10.3390/molecules21081100

**Published:** 2016-08-20

**Authors:** Shuang Ma, Patrick H. Toy

**Affiliations:** Department of Chemistry, University of Hong Kong, Pokfulam Road, Hong Kong, China; u3002732@connect.hku.hk

**Keywords:** self-supported catalyst, organocatalyst, *N*-heterocyclic carbene, benzoin condensation, redox esterification

## Abstract

The study of *N*-heterocyclic carbenes (NHCs) as organocatalysts has proliferated in recent years, and they have been found to be useful in a variety of reactions. In an attempt to further expand their utility and to study their recyclability, we designed and synthesized a series of self-supported NHCs in which the catalytic carbene groups form part of a densely functionalized polymer backbone, and studied them as organocatalysts. Of the self-Supported NHCs examined, a benzimidazole derived polymer with flexible linkers connecting the catalytic groups was found to be the most efficient organocatalyst in a model benzoin condensation reaction, and thus it was used in a variety of such reactions, including some involving catalyst recycling. Furthermore, it was also used to catalyze a set of redox esterification reactions involving conjugated unsaturated aldehydes. In all of these reactions the catalyst afforded good yield of the desired product and its polymeric nature facilitated product purification.

## 1. Introduction

**Figure 1:**
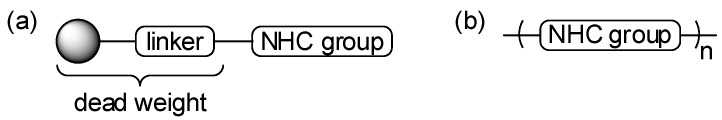
(**a**) Polymer-supported NHC organocatalysts. (**b**) Self-supported NHC organocatalysts.

As research regarding *N*-hereocyclic carbenes (NHCs) has increased in recent years, it has become clear that they are highly useful organocatalysts for a wide range of molecular transformations due to their ability to react with aldehydes and generate the corresponding homoenolate reactive intermediates [1,2,3,4,5,6,7,8,9,10,11,12]. In light of their great utility and specialized reactivity, efforts have been made to simplify their use and facilitate their recycling, and one strategy to achieve these aims has been to attach NHCs to a polymer support that allows for them to be easily removed at the end of a reaction and potentially reused. For example, imidazolium and related ionic liquids have been attached to various inert polymer carriers [13,14,15,16,17], and these can serve as protonated precursors to the corresponding NHC organocatalysts [18,19,20,21,22,23,24]. However, a drawback of this strategy is that the use of an inert polymer support lowers the loading level of the catalyst, even more so if a linker group is used for attachment, and increases the amount of solvent required for reactions (Figure 1a). An emerging strategy to work around this issue that potentially reduces the amount of dead weight, especially for imidazolium NHC precursors that require substituents on both nitrogen atoms (*vide infra*), is to self-support the catalyst by linking the catalytic groups in such a manner so that they are located in the main polymer chain, rather than attached to it (Figure 1b).

**Figure 2:**
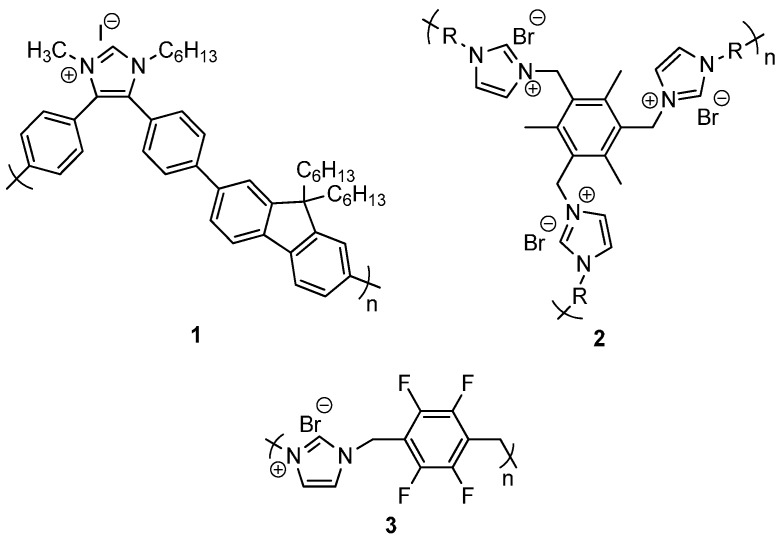
Examples of self-supported imidazolium salts used as *N*-hereocyclic carbene (NHC) precursors and catalysts.

For example, Cowley et al. described the use of **1** as a recyclable self-supported organocatalyst precursor for benzoin condensation reactions (Figure 2) [25]. However, relatively large solubilizing groups were used to link the imidazolium groups, which significantly lowered the catalytic group loading level of the polymer. Additionally, the linking groups were not attached to the nitrogen atoms, reducing the loading efficiency even further. In related research, Ying and co-workers used multifunctional linker groups to join imidazolium groups that did not bear solubilizing groups, and were attached at the nitrogen atoms to generate heterogeneous polymers, such as **2** [26,27,28,29]. These polymers were used as NHC precursors in catalytic applications, often in the presence of a metal such as Pd or Cu. Also, Liu et al. synthesized polymers, such as **3**, using fluorinated linker groups to connect imidazolium groups [30]. However, these polymers were not used as NHC precursors, but were directly applied as salts to catalyze CO_2_ cycloaddition reactions with epoxides to form cyclic carbonates.

Our group has had a longstanding interest in studying polymer-supported catalysts and reagents in general [31,32,33,34], and we have recently reported a self-supported oxime ligand for use in Pd-catalyzed Suzuki-Miyaura reactions [35]. This research has motivated us to examine self-supported imidazolium and benzimidazolium salts with flexible linker groups as NHC organocatalyt precursors. The premise underlying this research was that while the self-supported polymeric salts themselves would be insoluble in most organic solvents, the use of flexible linker groups would allow for the polymers to become soluble after deprotonation to generate the corresponding neutral self-supported NHC groups, which could then serve as homogeneous catalytic groups. Furthermore, it was expected that reprotonation of the polymers at the end of reactions would precipitate them from the reaction mixtures so that they could be separated from the desired products simply by filtration, and be ready for reuse. Herein we report the results of our research.

## 2. Results and Discussion

### 2.1. Synthesis of Self-Supported NHC Precursors

**Scheme 1:**
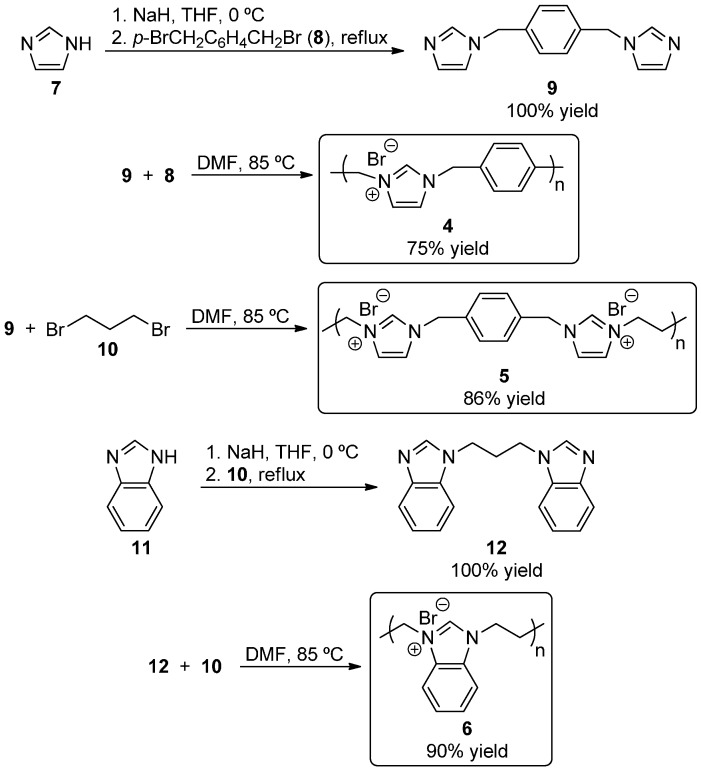
Synthesis of polymers **4**–**6**.

Since one of the major objectives of this research project was to examine what effect, if any, the use of flexible linker groups to connect the NHC groups has on their solubility, and thus catalytic utility, we targeted polymers **4**–**6** for study (Scheme 1), and methods similar to those utilized by Ying and co-workers were used for their preparation [26]. For the synthesis of **4** and **5**, two equivalents of imidazole (**7**) were deprotonated using NaH and then alkylated with a single equivalent of 1,4-bis(bromomethyl)benzene (**8**) to afford **9** in quantitative yield. Alkylation of **9** using **8** again in DMF at elevated temperature afforded polymeric imidazolium salt **4** in 75% yield. Using 1,3-dibromopropane (**10**) to alkylate **9** afforded polymer **5** in 86% yield. Similarly, two equivalents of benzimidazole (**11**) were alkylated as before with one equivalent of **10** to afford **12** in quantitative yield, and **12** was subsequently alkylated with **10** a second time to afford polymer **6** in 90% yield. Thus, polymers **4** (with relatively rigid linkers), **5** (with one relatively rigid linker and one flexible linker), and **6** (with two flexible linkers) were prepared in short order and high overall yield from simple starting materials.

### 2.2. Self-Supported NHCs as Catalysts in Benzoin Condensation Reactions

With our target polymeric salts in hand, we examined their utility as NHC precursors in the benzoin condensation of benzaldehyde (**13a**) to produce the alpha-hydroxy ketone product **14a**. The base DBU was used in DMSO for deprotonation of the polymers to generate the NHC catalytic groups, and in side-by-side reactions when no polymer or polymer **4** was used, no desired product **14a** was detected after 18 h (Table 1, entries 1 and 2). DBU was chosen as the base for these studies since it is typically used in such reactions, and we believe that the lack of reactivity of **4** was due to its complete insolublity. When polymers **5** and **6** were used, **14a** was formed in 74% and 82% isolated yield, respectively (entries 3 and 4). Thus, it does appear that incorporating flexible linker groups into the polymers does increase the catalytic utility of the self-supported NHC organocatalysts by increasing polymer solubility. While polymers **5** and **6** performed similarly, we chose to use **6** for our further studies. Changing the reaction solvent to THF resulted in significantly lower yield (entry 5), but using DMF allowed for high yield (84%) after only 5 h (entry 6). Increasing the reaction concentration further enhanced the isolated yield to 92% (entry 7). When no DBU was added, no reaction occurred, confirming the role of NHC catalysis (entry 8). For the sake of comparison, we prepared *N*,*N*’-diethylbenzi midazolium bromide (**15**, see Experimental Section) as a small molecule analogue of polymer **6**, and found that the corresponding NHC was much less effective as an organocatalyst (entry 9). Perhaps locating the NHC groups closely in a polymer allows for increased local concentration of the catalytic groups compared to the case of a molecular catalyst what would be more evenly distributed throughout the solution, and this high local concentration allows for synergism between the functional groups that results in improved catalytic efficiency [36,37].

Using the best identified reaction conditions, a variety of aromatic aldehydes were used as substrates in benzoin condensation reactions using polymer **6** as the precatalyst. As can be seen in Table 2, simple electron-donating and electron–withdrawing group substituted benzldehydes **13b**–**d** worked well in these reactions to produce the expected products **14b**–**d** in high isolated yields (entries 1–3). Highly electron-rich **13e** and highly electron-poor **13f** also worked well in these reactions, producing **14e** and **14f**, respectively (entries 5 and 6). Even napthaldehyde **13g** could be converted into product **14g** in excellent yield.

The possibility of recovering and reusing **6** was also explored using **13a** as the substrate (Table 3). After the reaction to form **13a** was complete, a solution of HCl in 1,4-dioxane (4.0 M) was added slowly dropwise into the mixture. The reaction mixture was stirred for an additional 1 h at room temperature, followed by the addition of ethyl acetate (20 mL). The liquid containing the crude **13a** was decanted and the light yellow precipitate **6** was washed with additional ethyl acetate (10 mL), and dried under vacuum (quantitative recovery). This was then directly used in the next reaction cycle. As can be seen in Table 3, polymer **6** was used five times with no obvious decrease in catalytic efficiency.

### 2.3. Self-Supported NHCs as Catalysts in Redox Esterification Reactions of α,β-Unsaturated Aldehydes

We next applied **6** in the redox esterification reactions of conjugated unsaturated aldehydes described by Chan and Scheidt [38]. Since it was previously reported that these reactions require a balance between the ability to generate the NHC catalyst through deprotonation of the benzimidazolium salt and the acidity needed to protonate the homoenolate equivalent [39], we examined the use of weakly acidic phenol additives in these reactions. Initial reactions were performed using cinnamaldehyde (**16A**) and benzyl alcohol (**17a**) as substrates (Table 4). When no additive was used, only 44% yield of ester **18Aa** was obtained in toluene at 100 °C after 4 h using 10 mol % **6** (entry 1). However, when PhOH (1 equiv.) was added and the reaction time was increased to 48 h, 86% of **18Aa** was obtained (entry 2). Switching to 4-nitrophenol (1 equiv.) further increased the yield of **18Aa** to 91% under otherwise identical reaction conditions (entry 3). Doubling the amount of **6** to 20 mol % allowed the reaction time to be shortened to 3 h (entry 4), but diluting the reaction only served to lower the yield and increase the reaction time necessary for completion (entry 5). Interestingly, using **15** in place of **6** lead to lowering of the isolated yield of **18Aa** to 51% even after 8 h.

Using the best identified reaction conditions we next examined the substrate scope of such redox esterification reaction using **6** as summarized in Table 5. Primary benzyl alcohols **17b**–**d** were reacted with **16A** to produce the corresponding esters **18Ab**–**d** in excellent yields (entries 1–3). When secondary benzyl alcohol **17e** was used, only low yield of **18Ae** was obtained (entry 4), as was the case with menthol (**17f**) to form **18Af** (entry 5). Perhaps not surprisingly, when no substrate alcohol was used, 4-nitrophenyl ester **18Ag** was obtained in very low yield (entry 6). Electron-withdrawing group substituted cinnamaldehydes **16B** and **16C** reacted with **17a** to form **18Ba** and **18Ca** in excellent yields, respectively (entries 7 and 8), as did electron-donating group substituted cinnamaldehydes **16D** and **16E** to form **18Da** and **18Ea**, respectively (entries 9 and 10). Even heterocyclic analogue of cinnamaldehyde **16F** produced the corresponding ester **18Fa** in very high yield (entry 11).

## 3. Experimental Section

### 3.1. General Information

Unless otherwise noted, all reagents were obtained from the Acros (Bridgewater, NJ, USA), Aldrich (St. Louis, MO, USA), or Alfa Aesar (Ward Hill, MA, USA) companies, and were used directly without purification. Toluene was dried using a Solv-Tek purification system employing activated Al_2_O_3_ (Innovative Technology China Ltd., Hong Kong, China). Tetrahedrofuran (THF) was distilled from Na metal under a N_2_ atmosphere before use. All reactions were carried out in dry glassware under a N_2_ atmosphere, and were monitored by TLC analysis using GF_254_ silica gel coated plates. The corresponding *R_f_* values and solvents used as eluents are listed. Column chromatography was performed with silica gel (230–400 mesh). NMR spectra were recorded on a Bruker (Billerica, MA, USA) DRX-300 or DRX-400 spectrometer operating at 300/400 MHz for ^1^H and 75/100 MHz for ^13^C analysis. Chemical shift data is expressed in ppm with reference to TMS. The following abbreviations were used for the assignment of the signals and their multiplicities: s (singlet), bs (broad singlet), d (doublet), t (triplet), q (quartet), p (pentet), m (multiplet), dt (double of triplet). Mass spectra data were obtained on a Finnigan MAT 96 mass spectrometer (Thermo Electron Corp., Madison, WI, USA).

### 3.2. Synthesis of NHC Precursors

Synthesis of **9** and **12**. Sodium hydride (60% in oil, 2.2 g, 55.0 mmol) was slowly added to imidazole (**7**, 3.5 g, 51.4 mmol) or benzimidazole (**11**, 6.0 g, 50.8 mmol) in dry THF (150 mL) at 0 °C, and the resulting suspension was stirred at rt for 2 h (until the hydrogen gas evolution ceased). Then **8** (for **9**, 13.2 g, 50.0 mmol) or **10** (for **12**, 10.1 g, 50 mmol) was added, and the resulting mixture was refluxed for 6 h more. The solvent was then removed under vacuum and the thus obtained crude product was dissolved in dichloromethane (150 mL) and washed with water (100 mL). The aqueous phase was extracted with dichloromethane (2 × 150 mL). The combined organic layers were washed with a saturated NaCl solution (200 mL), dried over anhydrous magnesium sulfate and filtered. After removal of the solvent under reduced pressure, the product was redissolved in dichloromethane (15 mL) and slowly added dropwise into diethyl ether (350 mL) to precipitate the pure product. The product **9** or **12** was dried at 70 °C under vacuum overnight and obtained in quantitative yield. See Supplementary Materials for NMR spectra.

*1,1′-(p-Phenylenedimethylene)bisimidazole* (**9**). White solid. ^1^H-NMR (400 MHz, CDCl_3_, ppm): δ 5.11 (s, 4H), 6.88 (s, 2H), 7.09 (s, 2H), 7.13 (s, 4H), 7.53 (s, 2H). ^13^C-NMR (100 MHz, CDCl_3_): δ 50.4, 119.3, 127.9, 130.1, 136.5, 137.5. LRMS for C_14_H_14_N_4_: calcd 238.1, found 238.0.

*1,1′-(1,3-Propanediyl)bis-1H-benzimidazole* (**12**). Light yellow solid. ^1^H-NMR (400 MHz, CDCl_3_, ppm): δ 2.56 (p, 2H, *J* = 4.0 Hz), 4.21 (t, 4H, *J* = 4.0 Hz), 7.277.34 (m, 6H), 7.84–7.86 (m, 2H), 7.87 (s, 2H). ^13^C-NMR (100 MHz, CDCl_3_): δ 29.4, 41.7, 109.3, 120.6, 122.5, 123.3, 133.3, 142.6, 143.9. LRMS for C_17_H_16_N_4_: calcd 276.1, found 276.0.

Synthesis of the polymeric salts **4**–**6**. The polymeric salts were made via direct alkylation of the **9** or **12** using **8** or **10** in a 1:1 molar ratio on a 10 mmol scale in dry DMF (150 mL). The reaction mixture was heated for two days at 85 °C. The solid product that precipitated from the solution and was filtered, washed with additional DMF (50 mL) and Et_2_O (2 × 50 mL), and dried under vacuum at 70 °C for 24 h. See Supplementary Materials for NMR spectra.

Polymer **4** was obtained in 75% yield as a white powder. It was completely insoluble in all solvents examined and thus was not characterized by NMR analysis.

Polymer **5** was obtained as light yellow powder with 86% yield. ^1^H-NMR (400 MHz, d_6_-DMSO, ppm): δ 2.42 (bs, 2H), 4.24–4.30 (m, 4H), 5.39–5.49 (m, 4H), 7.31–7.53 (m, 4H), 7.78–7.88 (m, 4H), 9.33–9.57 (m, 2H).

Polymer **6** was obtained as white powder with 90% yield. ^1^H-NMR (400 MHz, d_6_-DMSO, ppm): δ 2.67 (bs, 2H), 4.46–4.76 (m, 4H), 7.63–7.71 (m, 4H), 9.88–10.27 (m, 1H).

Synthesis of **15**. Sodium hydride (60% in oil, 2.2 g, 55.0 mmol) was added to a solution of **11** (6.0 g, 50.8 mmol) in THF (150 mL) at 0 °C and the resulting suspension was stirred at room temperature for 2 h (until hydrogen gas evolution ceased). Bromoethane (5.4 g, 50.0 mmol) was added to the reaction mixture and the resulting solution was heated at 70 °C for 6 h more. The solvent was removed under vacuum and the crude product was dissolved in dichloromethane (150 mL) and washed with water. The aqueous phase was extracted with dichloromethane (2 × 150 mL). The combined organic layers were washed with a saturated NaCl solution (200 mL), dried over anhydrous magnesium sulfate and filtered. The crude product was purified by silica gel chromatography to afford the desired product *N*-ethyl-*1H*-benzimidazole (93% yield) as yellow/brown oil. *N*-Ethyl-*1H*-benzimidazole (5.8 g, 40.0 mmol) and bromoethane (4.4 g, 40.0 mmol) were dissolved in 100 mL of THF. The reaction vial was sealed and the resulting solution was heated at 70 °C for 12 h. The slightly yellow solid product that precipitated from the solution was filtered, washed with diethyl ether (2 × 25 mL), and dried under vacuum at 70 °C for 24 h to afford **15** (95% yield). See Supplementary Materials for NMR spectra.

*1,3-Diethyl-1H-benzimidazolium bromide* (**15**). Pale yellow solid. ^1^H-NMR (400 MHz, CDCl_3_, ppm): δ 1.76 (t, 6H, *J* = 8.0 Hz), 4.71 (q, 4H, *J* = 8.0 Hz), 7.68–7.70 (m, 2H), 7.77–7.80 (m, 2H), 11.42 (s, 1H). ^13^C-NMR (100 MHz, CDCl_3_): δ 15.0, 43.0, 113.2, 127.3, 131.3, 142.2. LRMS for [C_11_H_15_N_2_]^+^: calcd 175.1, found 175.0.

### 3.3. General Procedure for the Benzoin Condensation Reactions

A Schlenk flask was charged with **13** (2.94 mmol) and **6** (10 mol %). Dry, degassed DMF (2 mL) was added via syringe, followed by DBU (15 mol %). The reaction mixture was stirred at room temperature under an atmosphere of nitrogen for the indicated time. Then a solution of HCl in dioxane (4.0 M, 0.2 mL) was added via syringe, and the reaction mixture was stirred for an additional 1 h at room temperature under nitrogen. Ethyl acetate (20 mL) was then added dropwise to precipitate the polymer. The liquid phase was decanted and the light yellow precipitate **6** was washed with additional ethyl acetate (10 mL), and dried under vacuum. The combined organic phase was then extracted into ethyl acetate (100 mL), washed with brine (3 × 100 mL) and the aqueous layer was back-extracted with ethyl acetate (100 mL). The organic layers were combined, dried with anhydrous magnesium sulfate, filtered, concentrated and purified by column chromatography (SiO_2_; hexanes: ethyl acetate). Characterization details for each compound are shown below. See Supplementary Materials for NMR spectra.

*2-Hydroxy-1,2-diphenylethanone* (**14a**). TLC (3:1 *v*/*v* hexanes:ethyl acetate): R_ƒ_ = 0.45. ^1^H-NMR (400 MHz, CDCl_3_, ppm): δ 4.56 (d, 1H, *J* = 8.0 Hz), 5.95 (d, 1H, *J* = 8.0 Hz), 7.24–7.36 (m, 4H), 7.39–7.44 (m, 2H), 7.52–7.58 (m, 1H), 7.89–7.93 (m, 2H). ^13^C-NMR (100 MHz, CDCl_3_): δ 76.4, 127.9, 128.7, 128.8, 129.2, 133.6, 134.0, 139.1, 199.1. LRMS for C_14_H_12_O_2_: calcd 212.1, found 212.1.

*2-Hydroxy-1,2-di(4-chloro-phenyl)ethanone* (**14b**). TLC (3:1 *v*/*v* hexanes:ethyl acetate): R_ƒ_ = 0.45. ^1^H-NMR (400 MHz, CDCl_3_, ppm): δ 4.52 (d, 1H, *J* = 8.0 Hz), 5.88 (d, 1H, *J* = 8.0 Hz), 7.23–7.26 (m, 2H), 7.30–7.32 (m, 2H), 7.37–7.40 (m, 2H), 7.81–7.84 (m, 2H). ^13^C-NMR (75 MHz, CDCl_3_): δ 75.6, 129.2, 129.3, 129.6, 130.6, 131.7, 134.9, 137.3, 140.9, 197.6. LRMS for C_14_H_10_Cl_2_O_2_: calcd 280.0, found 280.0.

*2-Hydroxy-1,2-di(4-bromo-phenyl)ethanone* (**14c**). TLC (3:1 *v*/*v* hexanes:ethyl acetate): R_ƒ_ = 0.45. ^1^H-NMR (400 MHz, CDCl_3_, ppm): δ 4.49 (d, 1H, *J* = 8.0 Hz), 5.86 (d, 1H, *J* = 8.0 Hz), 7.17–7.20 (m, 2H), 7.44–7.47 (m, 2H), 7.54–7.57 (m, 2H), 7.72–7.76 (m, 2H). ^13^C-NMR (100 MHz, CDCl_3_): δ 75.7, 129.5, 129.7, 130.6, 132.1, 132.3, 132.6, 137.8, 138.3, 197.8. LRMS for C_14_H_10_Br_2_O_2_: calcd 367.9, found 367.9.

*2-Hydroxy-1,2-di(4-methoxy-phenyl)ethanone* (**14d**). TLC (1:1 *v*/*v* hexanes:ethyl acetate): R_ƒ_ = 0.60. ^1^H-NMR (400 MHz, CDCl_3_, ppm): δ 3.73 (s, 3H), 3.80 (s, 3H), 4.55 (d, 1H, *J* = 8.0 Hz), 5.85 (d, 1H, *J* = 8.0 Hz), 6.87–6.83 (m, 4H), 7.27–7.23 (m, 2H), 7.92–7.88 (m, 2H). ^13^C-NMR (100 MHz, CDCl_3_): δ 55.7, 56.0, 75.7, 115.0, 126.9, 129.3, 131.7, 132.1, 160.1, 164.1, 197.6. LRMS for C_16_H_16_O_4_: calcd 272.1, found 272.1.

*2-Hydroxy-1,2-di(3,4,5-trimethoxy-phenyl)ethanone* (**14e**). TLC (1:5 *v*/*v* hexanes:ethyl acetate): R_ƒ_ = 0.56. ^1^H-NMR (300 MHz, CDCl_3_, ppm): δ 3.81 (s, 3H), 3.82–3.83 (m, 12H), 3.90 (m, 3H), 4.58 (d, 1H, *J* = 6.0 Hz), 5.77 (d, 1H, *J* = 6.0 Hz), 6.56 (s, 2H), 7.20 (s, 2H). ^13^C-NMR (75 MHz, CDCl_3_): δ 56.29, 56.31, 60.93, 61.09, 76.54, 104.83, 106.95, 128.31, 135.12, 153.07, 153.98, 197,43. LRMS for C_20_H_24_O_8_: calcd 392.1, found 392.2.

*2-Hydroxy-1,2-di(4-trifluoromethyl-phenyl)ethanone* (**14f**). TLC (3:1 *v*/*v* hexanes:ethyl acetate): R_ƒ_ = 0.22. ^1^H-NMR (400 MHz, CDCl_3_, ppm): δ 4.45 (bs, 1H), 6.03 (s, 1H), 7.46 (d, 2H, *J* = 8.0 Hz), 7.60 (d, 2H, *J* = 8.0 Hz), 7.69 (d, 2H, *J* = 8.0 Hz), 8.00 (d, 2H, *J* = 8.0 Hz). ^13^C-NMR (100 MHz, CDCl_3_): δ 76.1, 122.0, 124.7, 126.1, 126.4, 128.2, 129.5, 136.1, 138.3, 142.1, 197.8. LRMS for C_16_H_10_F_6_O_8_: calcd 348.1, found 348.1.

*2-Hydroxy-1,2-di(2-napthyl)ethanone* (**14g**). TLC (3:1 *v*/*v* hexanes:ethyl acetate): R_ƒ_ = 0.52. ^1^H-NMR (300 MHz, CDCl_3_, ppm): δ 4.83 (s, 1H), 6.31 (s, 1H), 7.42–7.57 (m, 5H), 7.75–8.03 (m, 8H), 8.52 (s, 1H), 7.72–7.76 (m, 2H). ^13^C-NMR (75 MHz, CDCl_3_): δ 76.5, 124.3, 125.0, 126.5, 126.6, 127.0, 127.6, 127.78, 127.81 128.1, 128.7, 129.1, 129.2, 129.8, 130.9, 131.5, 132.3, 133.3, 133.5, 135.9, 136.7, 199.0. LRMS for C_22_H_16_O_2_: calcd 312.1, found 312.1.

### 3.4. Recycling of Polymer ***5*** from Benzoin Condensation Reactions

After the reaction was complete, the precipitated polymer was separated as above, and the light yellow polymer was washed with additional ethyl acetate (10 mL) and dried under vacuum (quantitative recovery of **6**), and used directly for the next reaction cycle.

### 3.5. General Procedure for the Redox Esterification of α,β-Unsaturated Aldehydes

Aldehyde **16** (1.5 mmol), alcohol **17** (3.0 mmol), and **6** (20 mol %) were mixed in toluene (1 mL) in a reaction vial. The vial was flushed with N_2_. DBU (25 mol %) was then added slowly dropwise into the reaction vial and the reaction mixture, followed by the addition of 4-nitrophenol (100 mol %), the reaction mixture was then stirred at 100 °C. The reaction was monitored by TLC and stirred for time indicated in Table 5. The mixture was then cooled to room temperature and the reaction was quenched by a solution of HCl in 1,4-dioxane (4.0 M, 0.2 mL). Ethyl acetate (20 mL) was dropwise into the vial to precipitate the polymeric catalyst. The precipitate was filtered and the filtrate was washed with additional ethyl acetate (10 mL). The combined organic phase was concentrated under vaccum and purified by column chromatography (SiO_2_; hexanes: ethyl acetate). Characterization details for each compound are shown below. See Supplementary Materials for NMR spectra.

*Benzyl 3-phenylpropanoate* (**18Aa**). TLC (1:20 *v*/*v* hexanes:ethyl acetate): R_ƒ_ = 0.52. ^1^H-NMR (300 MHz, CDCl_3_, ppm): δ 2.67 (t, 2H, *J* = 7.5 Hz), 2.96 (t, 2H, *J* = 7.5 Hz), 7.15–7.39 (m, 10H). ^13^C-NMR (75 MHz, CDCl_3_): δ 31.0, 36.0, 66.3, 118.0, 128.6, 128.7, 129.0, 130.4, 136.0, 140.5, 145.3, 172.8. LRMS for C_14_H_12_O_2_: calcd 240.1, found 240.1.

*4-Bromobenzyl 3-phenylpropanoate* (**18Ab**). TLC (1:20 *v*/*v* hexanes:ethyl acetate): R_ƒ_ = 0.45. ^1^H-NMR (300 MHz, CDCl_3_, ppm): δ 2.68 (t, 2H, *J* = 7.5 Hz), 2.94 (t, 2H, *J* = 7.5 Hz), 5.04 (s, 2H), 7.14–7.50 (m, 9H). ^13^C-NMR (75 MHz, CDCl_3_) δ 31.0, 35.9, 65.6, 122.4, 126.5, 128.4, 128.7, 130.0, 131.8, 135.1, 140.4, 172.7. HRMS for C_16_H_15_BrO_2_: calcd 318.0255, found 318.0245.

*2-Bromobenzyl 3-phenylpropanoate* (**18Ac**). TLC (1:20 *v*/*v* hexanes:ethyl acetate): R_ƒ_ = 0.50. ^1^H-NMR (300 MHz, CDCl_3_, ppm): δ 2.76 (t, 2H, *J* = 7.5 Hz), 3.04 (t, 2H, *J* = 7.5 Hz), 5.23 (s, 2H), 7.17–7.34 (m, 8H), 7.60 (d, 1H, *J* = 9.0 Hz). ^13^C-NMR (75 MHz, CDCl_3_) δ 31.0, 35.8, 65.9, 123.5, 126.4, 127.5, 128.4, 128.6, 129.7, 129.9, 132.9, 135.3, 140.4, 172.6. HRMS for C_16_H_15_BrO_2_: calcd 318.0255, found 318.0245.

*2-Chlorobenzyl 3-phenylpropanoate* (**18Ad**). TLC (1:20 *v*/*v* hexanes:ethyl acetate): R_ƒ_ = 0.50. ^1^H-NMR (300 MHz, CDCl_3_, ppm): δ 2.72 (t, 2H, *J* = 7.5 Hz), 2.99 (t, 2H, *J* = 7.5 Hz), 5.22 (s, 2H), 7.19–7.40 (m, 9H). ^13^C-NMR (75 MHz, CDCl_3_) δ 31.0, 35.9, 63.7, 126.4, 126.9, 128.2, 128.4, 128.6, 129.0, 129.5, 129.9, 133.7, 140.4, 172.6. HRMS for C_16_H_15_BrO_2_: calcd 274.0761, found 274.0759.

*1-Phenylethyl 3-phenylpropanoate* (**18Ae**). TLC (1:20 *v*/*v* hexanes:ethyl acetate): R_ƒ_ = 0.50. ^1^H-NMR (300 MHz, CDCl_3_, ppm): δ 1.49 (d, 3H, *J* = 6.0 Hz), 2.64 (t, 2H, *J* = 7.5 Hz), 2.94 (t, 2H, *J* = 7.5 Hz), 5.88 (q, 1H, *J* = 6.0 Hz), 7.15–7.35 (m, 10H). ^13^C-NMR (75 MHz, CDCl_3_) δ 22.2, 31.0, 36.2, 72.4, 126.2, 126.3, 127.9, 128.4, 128.6, 140.5, 141.7, 172.2. LRMS for C_17_H_13_O_2_: calcd 254.1, found 254.1.

*2-Isopropyl-4-methyl-cyclohexyl 3-phenylpropanoate* (**18Af**). TLC (1:20 *v*/*v* hexanes:ethyl acetate): R_ƒ_ = 0.56. ^1^H-NMR (400 MHz, CDCl_3_, ppm): δ 0.69 (d, 3H, *J* = 4.0 Hz), 0.80–0.93 (m, 8H), 1.25–1.36 (m, 1H), 1.41–1.52 (m, 1H), 1.61–1.76 (m, 4H), 1.91–1.95 (m, 1H), 2.58–2.62 (m, 2H), 2.92–2.96 (m, 2H), 4.67 (dt, 1H, *J* = 12.0 Hz), 7.18–7.28 (m, 5H). ^13^C-NMR (100 MHz, CDCl_3_): δ 16.4, 20.9, 22.1, 23.5, 26.3, 31.2, 31.5, 34.4, 36.3, 41.0, 47.1, 74.3, 126.3, 128.4, 128.5, 140.7, 172.6. HRMS for C_19_H_28_O_2_: calcd 288.2089, found 288.2082.

*4-Nitrophenyl-3-phenylpropanoate* (**18Ag**). TLC (1:20 *v*/*v* hexanes:ethyl acetate): R_ƒ_ = 0.50. ^1^H-NMR (400 MHz, CDCl_3_): δ 2.94 (t, 2H, *J* = 8.0 Hz), 3.09 (t, 2H, *J* = 8.0 Hz), 7.17–7.20 (m, 2H), 7.26–7.27 (m, 2H), 7.32–7.36 (m, 2H), 8.24–8.26 (m, 2H, *J* = 7.5 Hz). ^13^C-NMR (CDCl_3_, 100 MHz): 30.1, 35.6, 120.5, 122.3, 125.2, 130.1, 131.7, 139.0, 145.3, 155.5, 170.3. LRMS for C_15_H_13_NO_4_: calcd 271.1, found 271.1.

*Benzyl 3-(4-bromophenyl)propanoate* (**18Ba**). TLC (1:20 *v*/*v* hexanes:ethyl acetate): R_ƒ_ = 0.38. ^1^H-NMR (300 MHz, CDCl_3_, ppm): δ 2.68 (t, 2H, *J* = 9.0 Hz), 2.95 (t, 2H, *J* = 7.5 Hz), 5.11 (s, 2H), 7.11–7.14 (m, 2H), 7.29–7.35 (m, 6H). ^13^C-NMR (75 MHz, CDCl_3_): δ 30.36, 35.65, 66.40, 120.14, 128.30, 128.32, 128.61, 130.17, 131.59, 135.85, 139.38, 172.40. LRMS for C_16_H_15_BrO_2_: calcd 318.0, found 318.0.

*Benzyl 3-(4-cyanophenyl)propanoate* (**18Ca**). TLC (1:15 *v*/*v* hexanes:ethyl acetate): R_ƒ_ = 0.40. ^1^H-NMR (400 MHz, CDCl_3_, ppm): δ 2.69 (t, 2H, *J* = 8.0 Hz), 3.02 (t, 2H, *J* = 6.0 Hz), 5.10 (s, 2H), 7.26–7.29 (m, 4H), 7.33–7.36 (m, 2H), 7.63–7.55 (m, 2H). ^13^C-NMR (100 MHz, CDCl_3_): δ 30.8, 35.0, 66.4, 110.1, 118.9, 128.2, 128.3, 128.5, 129.1, 132.2, 135.6, 145.9, 171.9. HRMS for C_17_H_15_O_2_N: calcd 265.1103, found 265.1101.

*Benzyl 3-(4-tert-butylphenyl)propanoate* (**18Da**). TLC (1:20 *v*/*v* hexanes:ethyl acetate): R_ƒ_ = 0.42. ^1^H-NMR (300 MHz, CDCl_3_, ppm): δ 2.68 (t, 2H, *J* = 9.0 Hz), 2.95 (t, 2H, *J* = 7.5 Hz), 5.11 (s, 2H), 7.11–7.14 (m, 2H), 7.29–7.35 (m, 6H). ^13^C-NMR (75 MHz, CDCl_3_): δ 30.5, 31.3, 31.5, 36.0, 66.4, 125.5, 128.1, 128.3, 128.6, 136.1, 137.4, 149.2, 173.0. HRMS for C_20_H_24_O_2_: calcd 296.1776, found 296.1174.

*Benzyl 3-(4-methoxyphenyl)propanoate* (**18Ea**). TLC (1:15 *v*/*v* hexanes:ethyl acetate): R_ƒ_ = 0.55. ^1^H-NMR (300 MHz, CDCl_3_, ppm): δ 2.65 (t, 2H, *J* = 7.5 Hz), 2.91 (t, 2H, *J* = 7.5 Hz), 3.78 (s, 3H), 5.10 (s, 2H), 6.82 (d, 2H, *J* = 9.0 Hz), 7.09 (d, 2H, *J* = 9.0 Hz), 7.29–7.36 (m, 5H). ^13^C-NMR (75 MHz, CDCl_3_): δ 30.2, 36.2, 55.3, 66.3, 113.9, 128.3, 128.6, 129.3, 132.5, 135.8, 158.1, 172.8. LRMS for C_17_H_16_O_3_: calcd 270.1, found 270.0.

*Benzyl 3-(furan-2-yl)propanoate* (**18Fa**). TLC (1:20 *v*/*v* hexanes:ethyl acetate): R_ƒ_ = 0.36. ^1^H-NMR (400 MHz, CDCl_3_, ppm): δ 2.68 (t, 2H, *J* = 8.0 Hz), 2.97 (t, 2H, *J* = 8.0 Hz), 5.11 (s, 2H), 5.98–5.99 (m, 1H), 6.23–6.24 (m, 1H), 7.26–7.35 (m, 6H). ^13^C-NMR (100 MHz, CDCl_3_): δ 23.5, 32.7, 66.4, 105.4, 110.0, 110.2, 128.3, 128.6, 135.9, 141.3, 154.1, 172.3. LRMS for C_14_H_14_O_3_: calcd 230.1, found 210.1.

## 4. Conclusions

In conclusion, we have prepared a series of self-supported imidazolium and benzimidazolium polymeric salts (**4**–**6**) that can function as NHC precursors by attaching the linker groups as nitrogen substituents. These were heterogeneous at the outset of reactions, but when deprotonated in situ using DBU to generate the corresponding NHC organocatalytic groups, they became soluble, and were examined in benzoin condensation reactions. Polymer **6** performed best in the model reaction and it was used in a range of benzoin condensation and conjugated unsaturated aldehyde redox esterification reactions. Importantly polymer **6** could be recovered and reused from the former reactions by protonation to regenerate and precipitate it with no apparent decrease in utility through five reaction cycles. Furthermore, it appeared that the self-supported catalytic groups exhibited synergistic effects that were not observed when analogous molecular catalyst precursor **14** was used. Further study of this observation is currently ongoing, as is identification of additional applications for **6**. Results of this research will be reported shortly.

## Figures and Tables

**Table 1:**
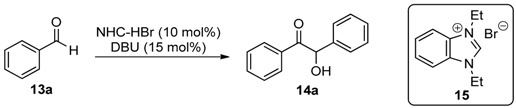
Benzoin condensation reaction of **13a** using polymers **4**–**6** and **15** ^a^.

Entry	NHC-HBr	Solvent	Time (h)	Isolated Yield (%)
1	--	DMSO (2 mL)	18	0
2	4	DMSO (2 mL)	18	0
3	5	DMSO (2 mL)	18	74
4	6	DMSO (2 mL)	18	82
5	6	THF (2 mL)	18	41
6	6	DMF (2 mL)	5	84
7	6	DMF (1 mL)	5	92
8	6	DMF (1 mL) ^b^	5	0
9	15	DMF (1 mL)	5	7

^a^ Unless otherwise specified, the reactions were performed on a 0.5 mmol scale of benzaldehyde with 10 mol % NHC precatalysts and 15 mol % DBU at room temperature; ^b^ Without DBU.

**Table 2:**
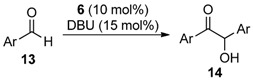
Substrate scope of benzoin condensation reactions using polymer **6** ^a^.

Entry	Substrate	Time (h)	Product	Isolated Yield (%)
1	Ar = 4-Cl-C_6_H_4_- (**13b**)	12	Ar = 4-Cl-C_6_H_4_- (**14b**)	94
2	Ar = 4-Br-C_6_H_4_- (**13c**)	12	Ar = 4-Br-C_6_H_4_- (**14c**)	88
3	Ar = 4-MeO-C_6_H_4_- (**13d**)	12	Ar = 4-MeO-C_6_H_4_- (**14d**)	82
4	Ar = 3,4,5-(MeO)_3_ -C_6_H_2_- (**13e**)	5	Ar = 3,4,5-(MeO)_3_ -C_6_H_2_- (**14e**)	82
5	Ar = 4-CF_3_-C_6_H_4_- (**13f**)	12	Ar = 4-CF_3_-C_6_H_4_- (**14f**)	78
6	Ar = 2-Napth- (**13g**)	5	Ar = 2-Napth- (**14g**)	88

^a^ The reactions were performed on a 0.5 mmol scale of aldehyde with 10 mol % **5** and 15 mol % DBU at room temperature in 1 mL dry DMF.

**Table 3:**
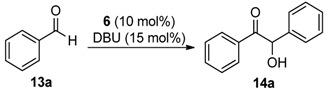
Recycling experiments using polymer **6** ^a^.

Cycle	1	2	3	4	5
Isolated Yield (%)	92	92	91	90	92

^a^ The reactions were performed on a 0.5 mmol scale of **12a** with 10 mol % **6** precatalysts and 15 mol % DBU at room temperature in 1 mL dry DMF.

**Table 4:**
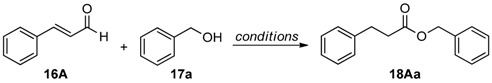
NHC-catalyzed redox esterification reactions using polymer **6** ^a^.

Entry	6 (mol %)	Additive (1 equiv.)	Time (h)	Isolated Yield (%)
1	10	--	4	44
2	10	phenol	48	86
3	10	4-nitrophenol	48	91
4	20	4-nitrophenol	3	90
5 ^b^	20	4-nitrophenol	8	72
6 ^c^	20	4-nitrophenol	8	51

^a^ Unless otherwise specified, the reactions were performed on a 1.5 mmol of **16A** and 3.0 mmol of **17a** with a 1:1.25 molar ratio of **6** to DBU in 1 mL toluene; ^b^ 2 mL toluene was used; ^c^ **15** was used as the precatalyst instead of **6**.

**Table 5:**
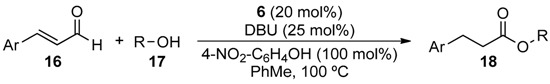
Substrate scope of redox esterification reactions using **6** ^a^.

Entry	Ar	R	Time (h)	Product	Isolated Yield (%)
1	Ph- (16A)	4-Br-C_6_H_4_CH_2_- (**17b**)	7	**18Ab**	94
2	Ph- (16A)	2-Br-C_6_H_4_CH_2_- (**17c**)	12	**18Ac**	92
3	Ph- (16A)	2-Cl-C_6_H_4_CH_2_- (**17d**)	12	**18Ad**	94
4	Ph- (16A)	PhCH(CH_3_)- (**17e**)	20	**18Ae**	30
5	Ph- (16A)	menthyl- (**17f**)	48	**18Af**	30
6 ^b^	Ph- (16A)	--	48	**18Ag**	7
7	4-Br-C_6_H_4_- (16B)	Bn- (**17a**)	4	**18Ba**	89
8	4-CN-C_6_H_4_- (16C)	Bn- (**17a**)	3	**18Ca**	89
9	4-tBu-C_6_H_4_- (16D)	Bn- (**17a**)	3	**18Da**	90
10	4-MeO-C_6_H_4_- (16E)	Bn- (**17a**)	5	**18Ea**	79
11	2-furanyl- (16F)	Bn- (**17a**)	7	**18Fa**	89

^a^ Unless otherwise specified, the reactions were performed on a 1.5 mmol scale of aldehyde **16** with 2.0 equiv of alcohol **17**, 20 mol % **6** and 25 mol % DBU at 100 °C in 1 mL toluene; ^b^ Product (**18Ag**) formed from the reaction of cinnamaldehyde and 4-nitrophenol.

## Supplementary Materials

Copies of the NMR spectra can be accessed at: http://www.mdpi.com/1420-3049/21/8/1100/s1.
