# Supplementary material for: Self-Supported N-Heterocyclic Carbenes and Their Use as Organocatalysts
Source: Molecules. 2016 Aug 20;21(8):1100. doi: 10.3390/molecules21081100 (PMC6274222; doi:10.3390/molecules21081100)
Supplement: Supplementary file 1 [file molecules-21-01100-s001.pdf]

Supplementary Materials: Self-Supported N-Heterocyclic Carbenes and Their Use as Organocatalysts

Shuang Ma and Patrick H. Toy

Contents

NMR Spectra of NHC precursors S**2**

NMR Spectra of benzoin condensation reaction products S**6**

NMR Spectra of redox esterification reaction products S**12**

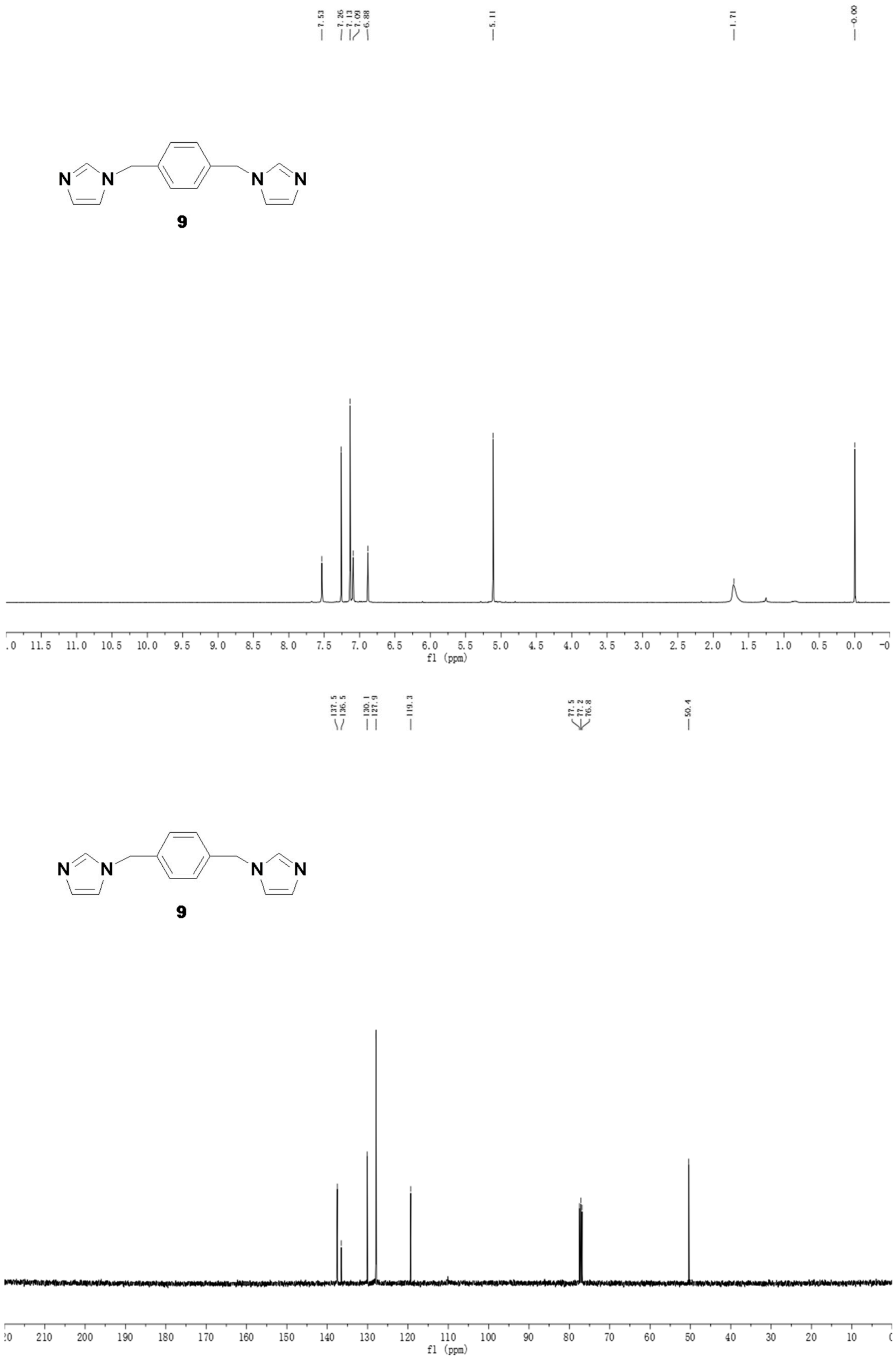

Figure S1. ^{1}H- and ^{13}C-NMR spectra for **9**.

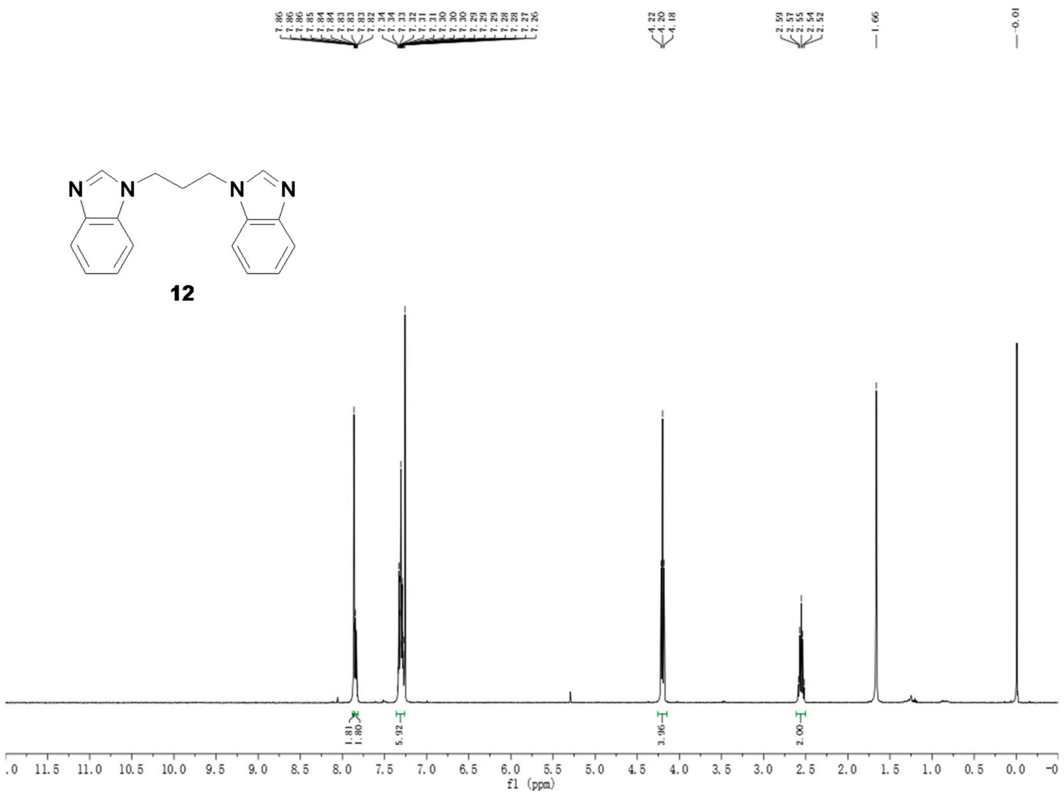

12

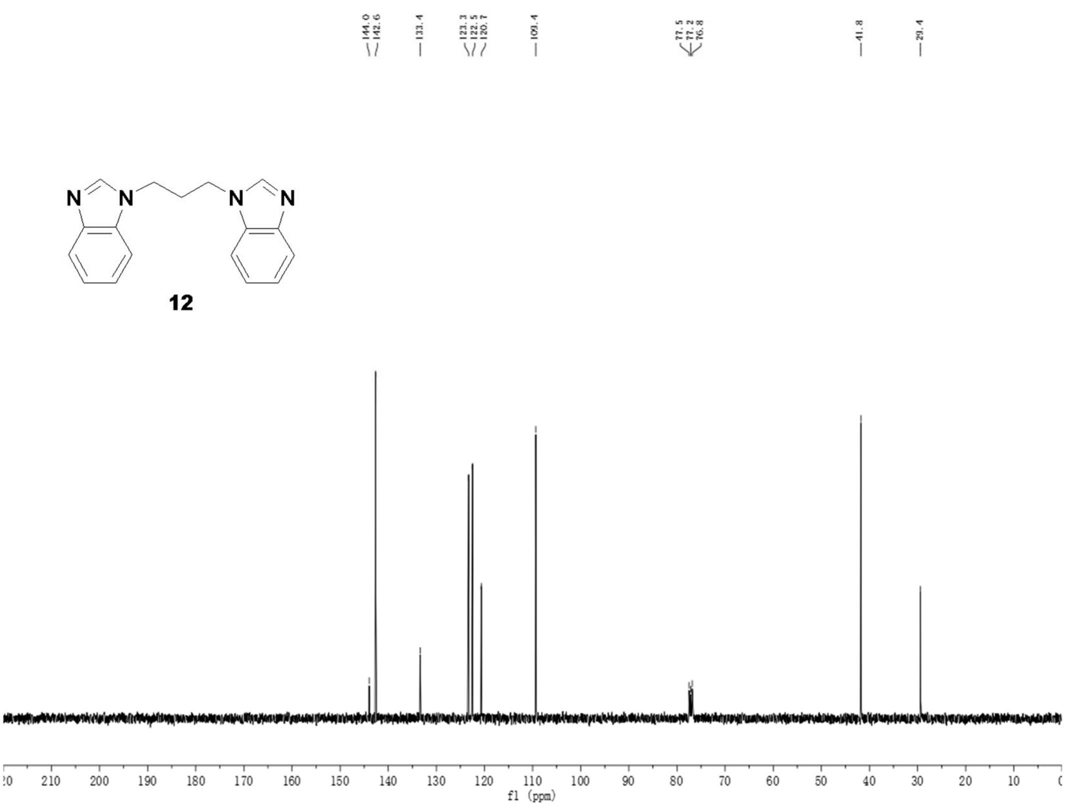

12

Figure S2. ^{1}H- and ^{13}C-NMR spectra for **12**.

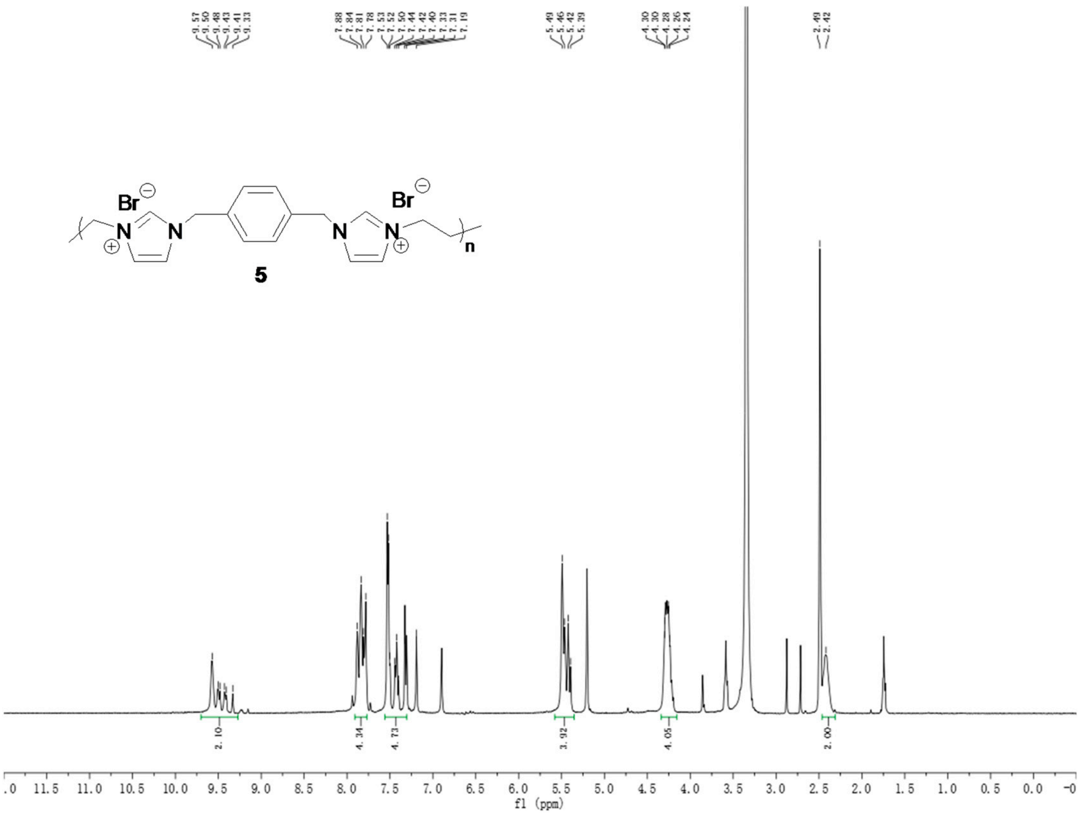

Figure S3. ^{1}H-NMR spectrum for **5**.

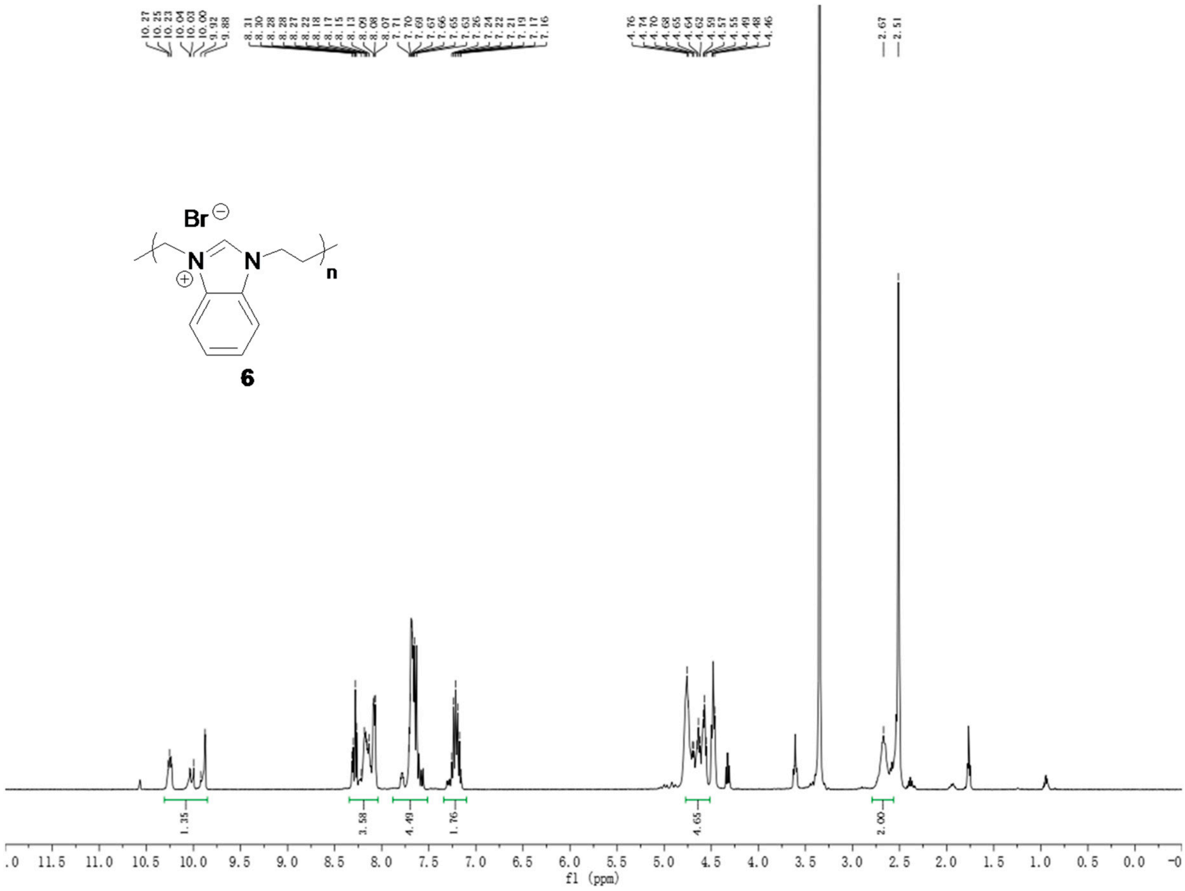

Figure S4. ^{1}H-NMR spectrum for **6**.

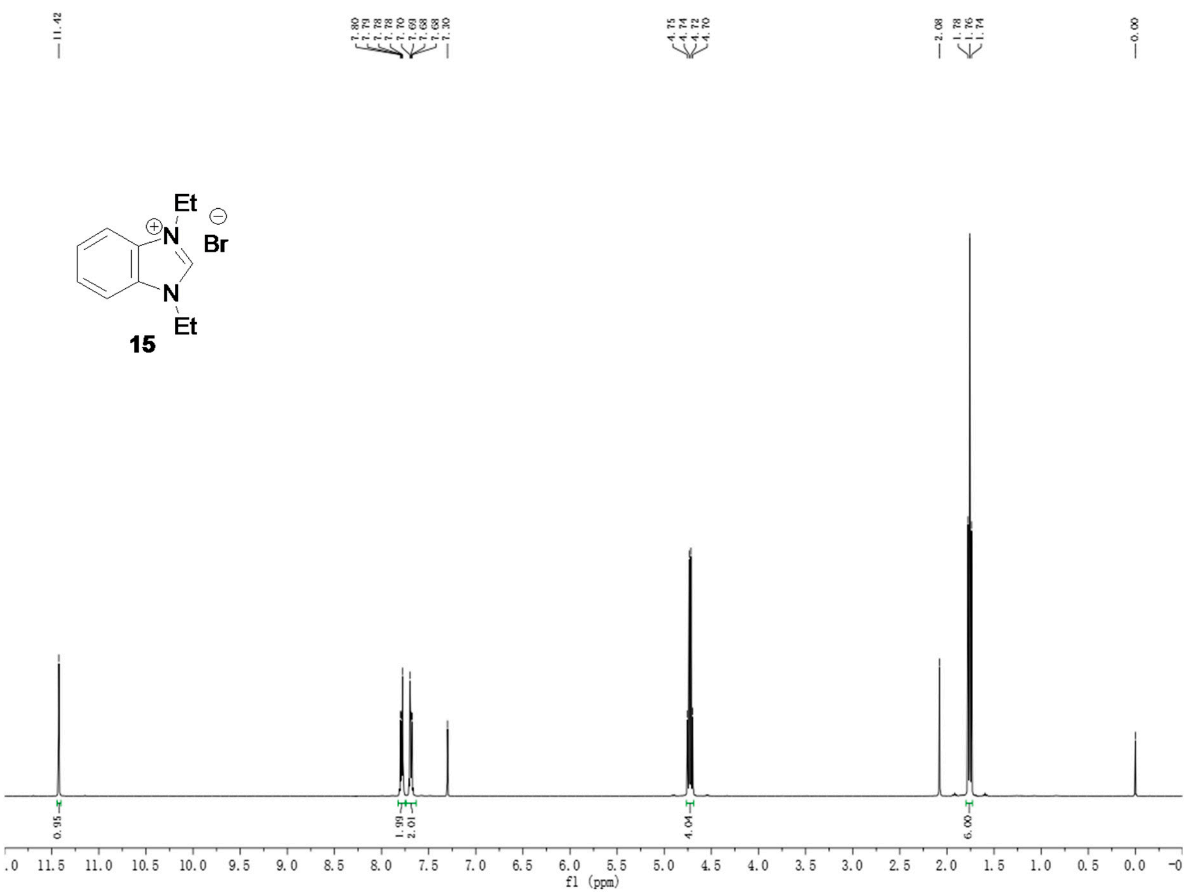

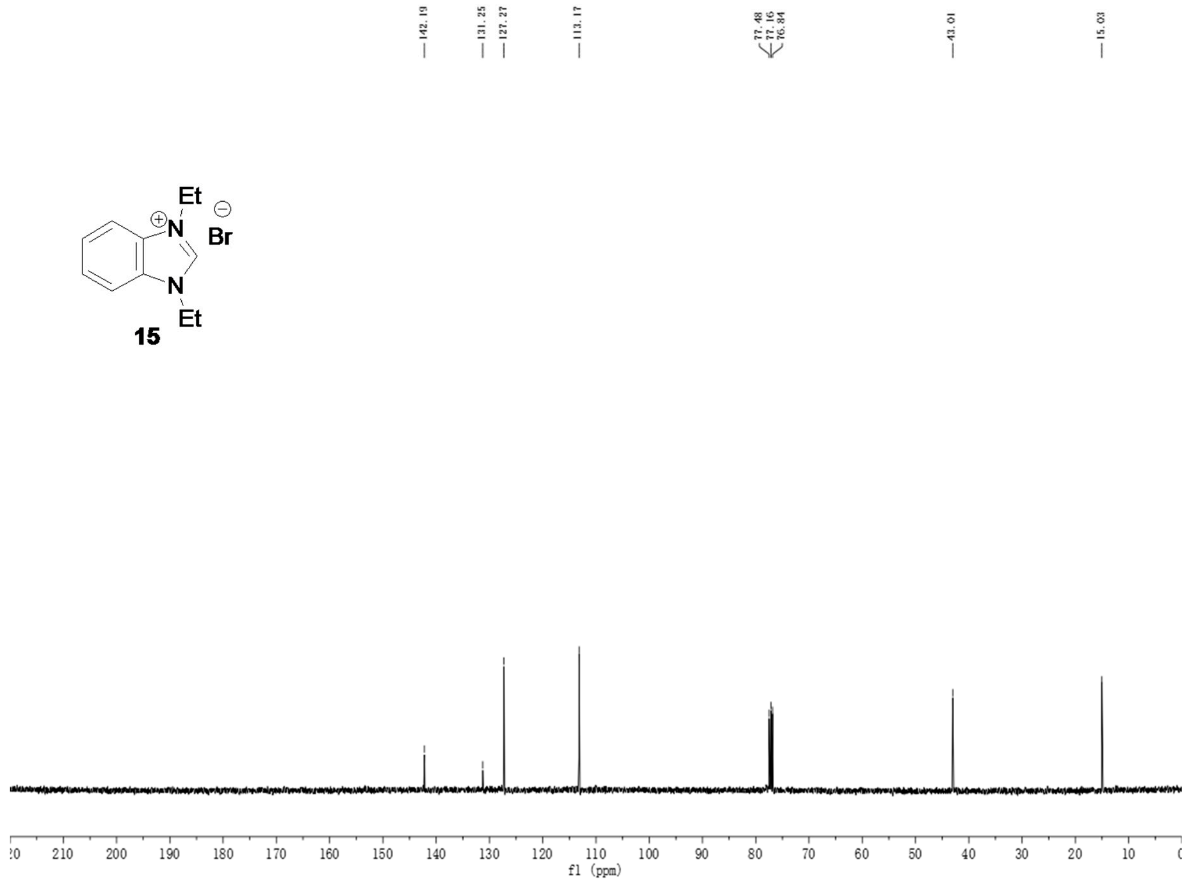

Figure S5. ^{1}H- and ^{13}C-NMR spectra for **15**.

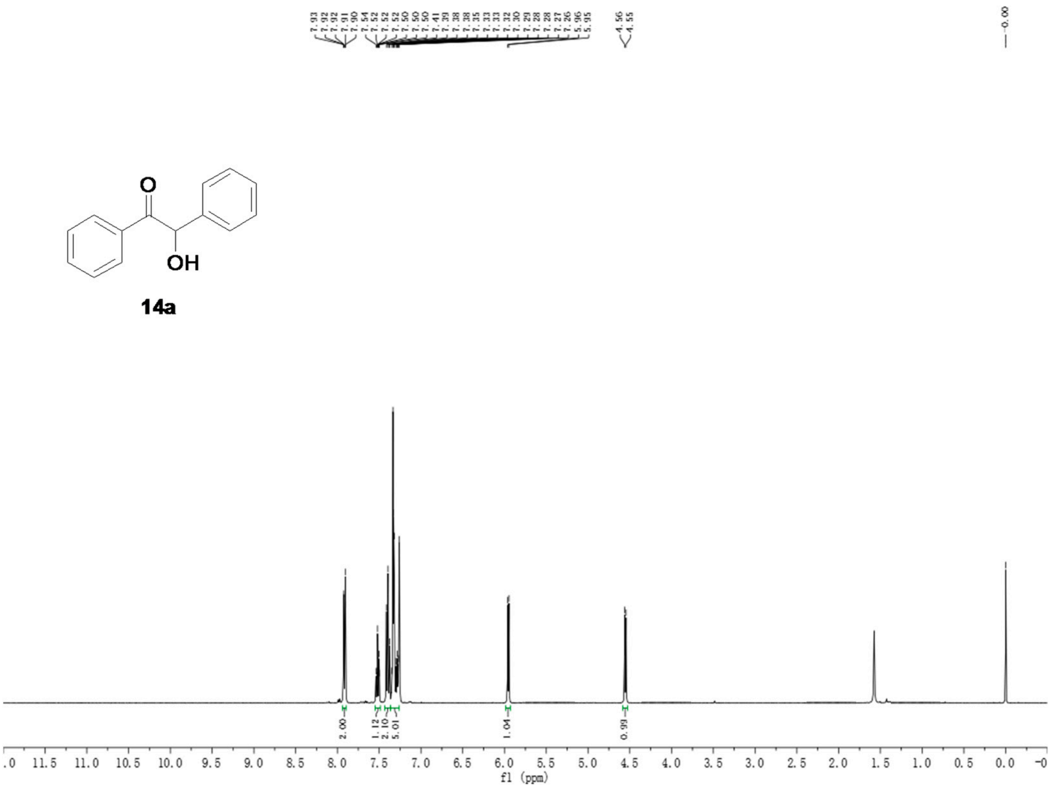

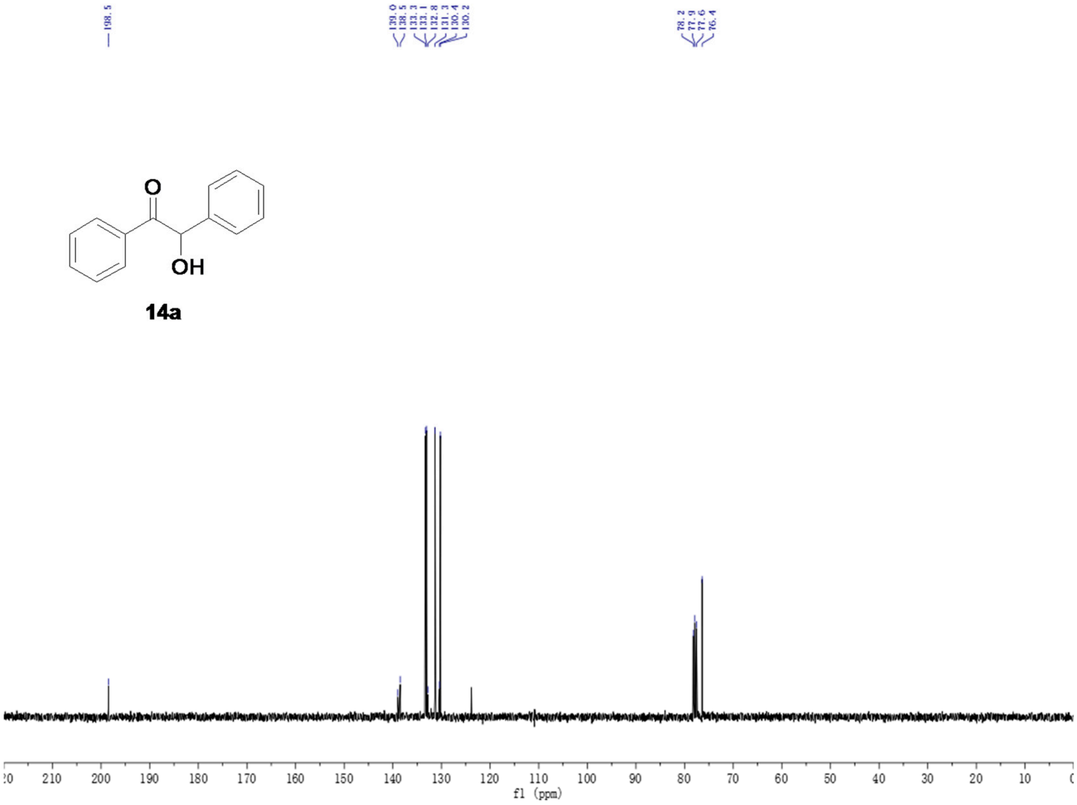

Figure S6. ^{1}H- and ^{13}C-NMR spectra for **14a**.

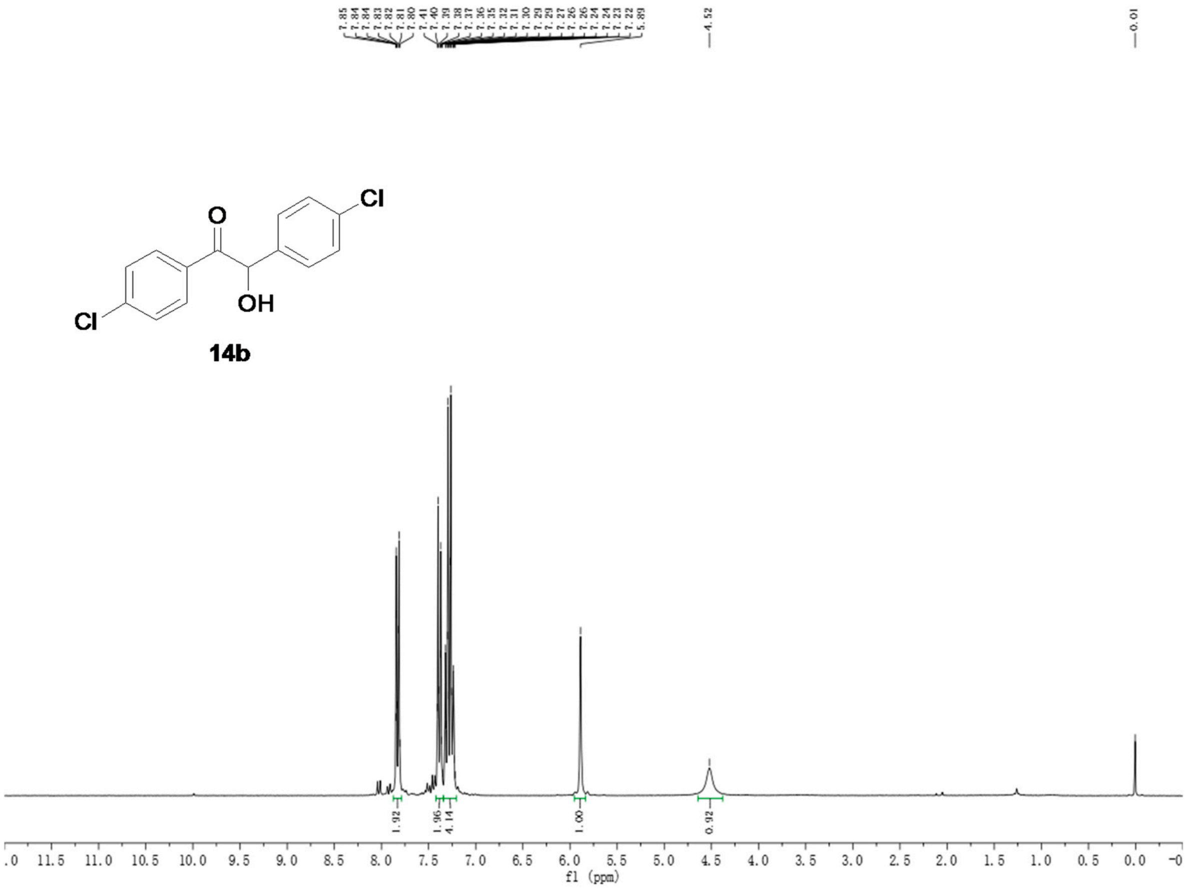

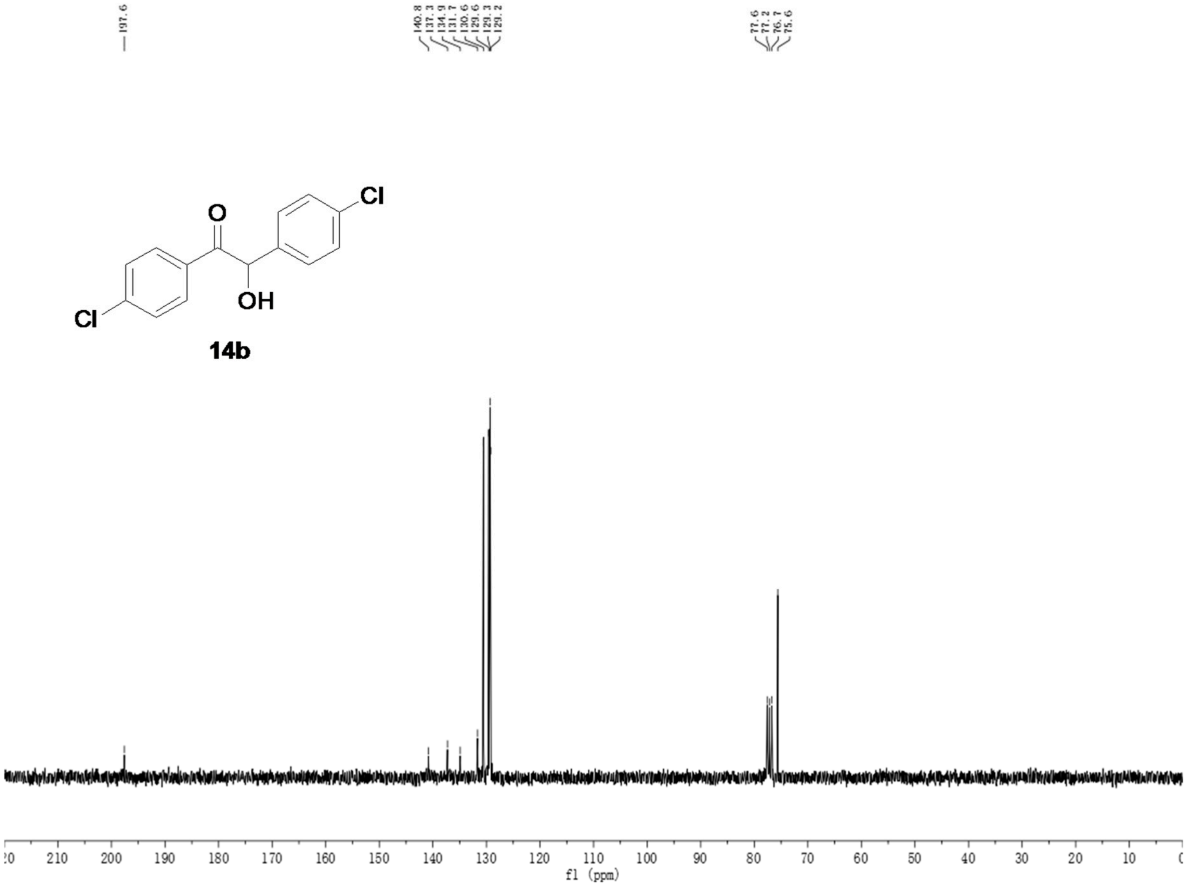

Figure S7. ^{1}H- and ^{13}C-NMR spectra for **14b**.

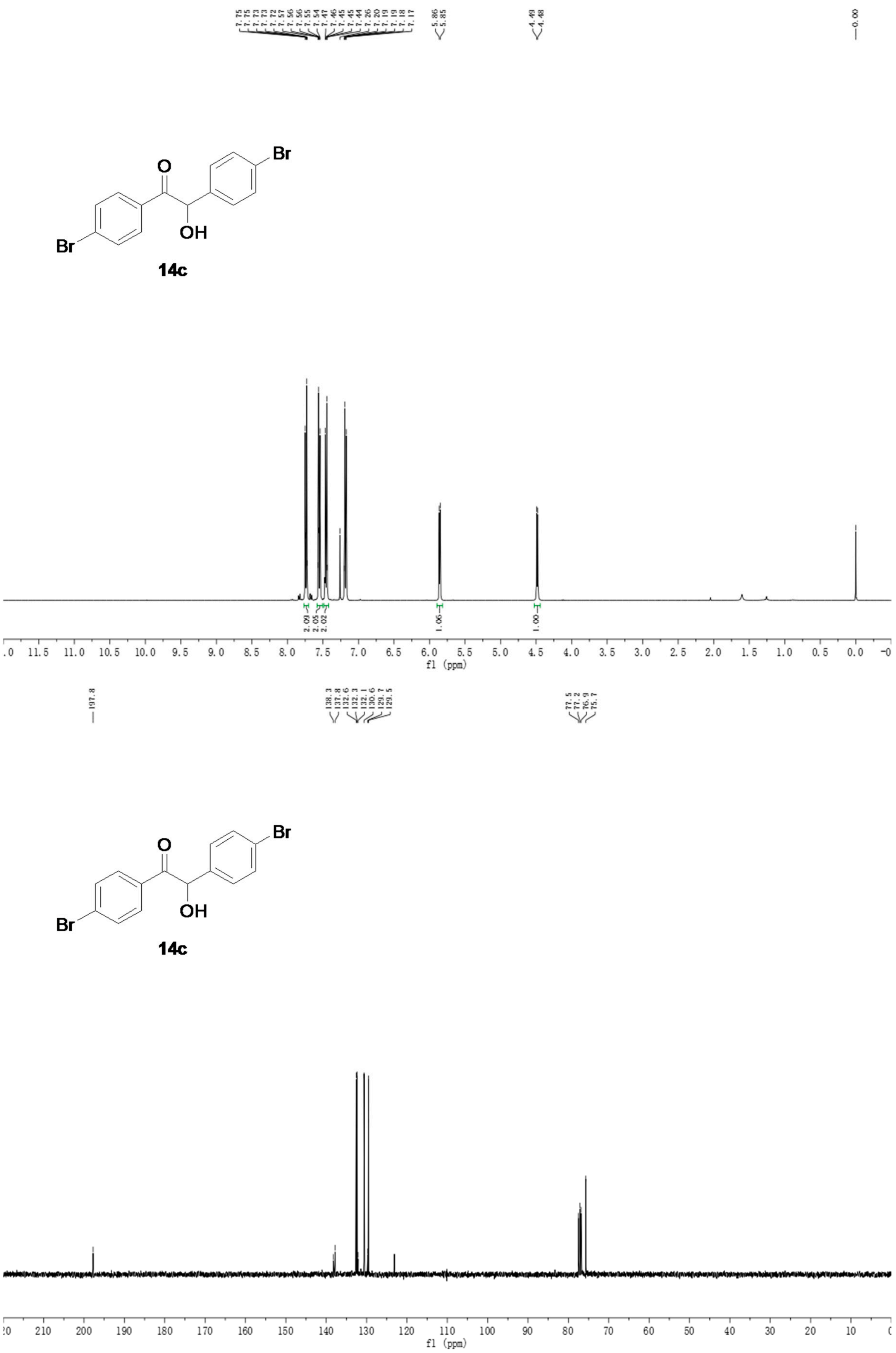

Figure S8. ^{1}H- and ^{13}C-NMR spectra for **14c**.

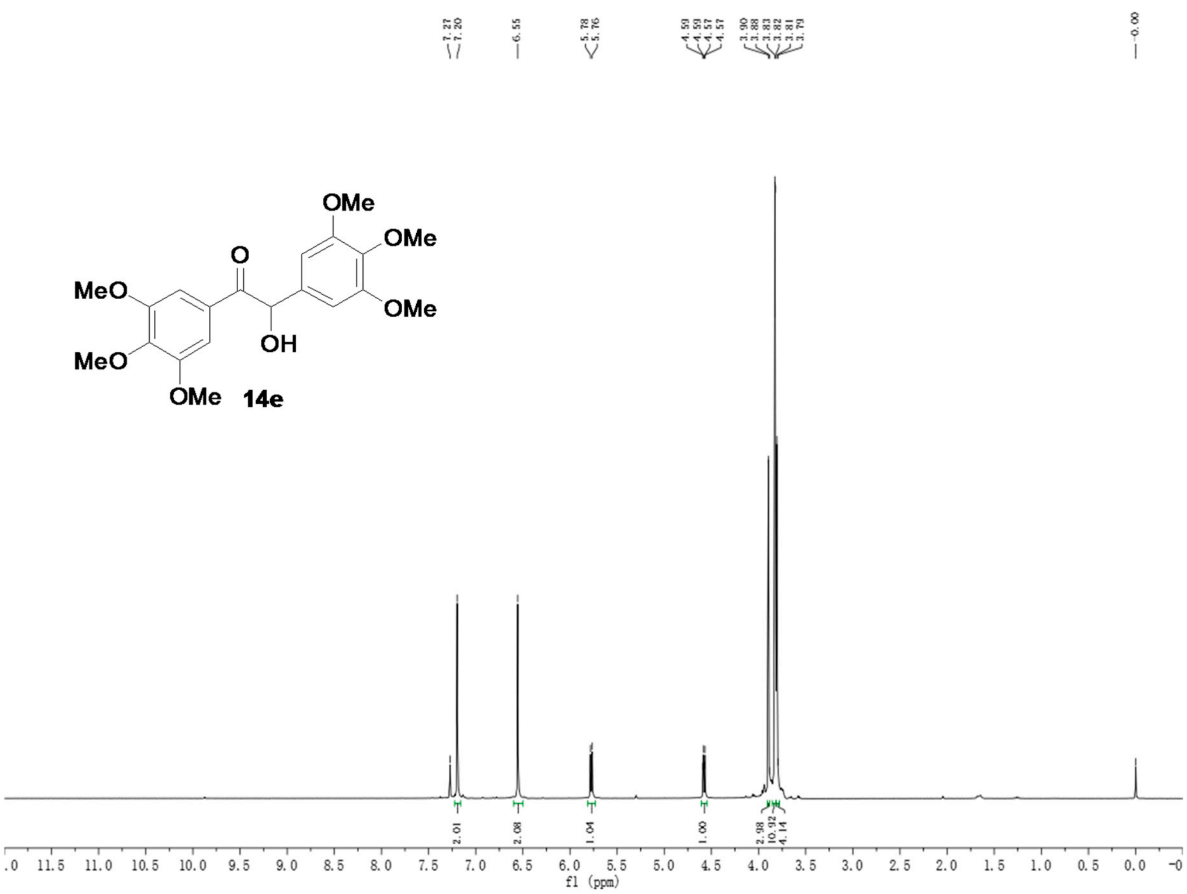

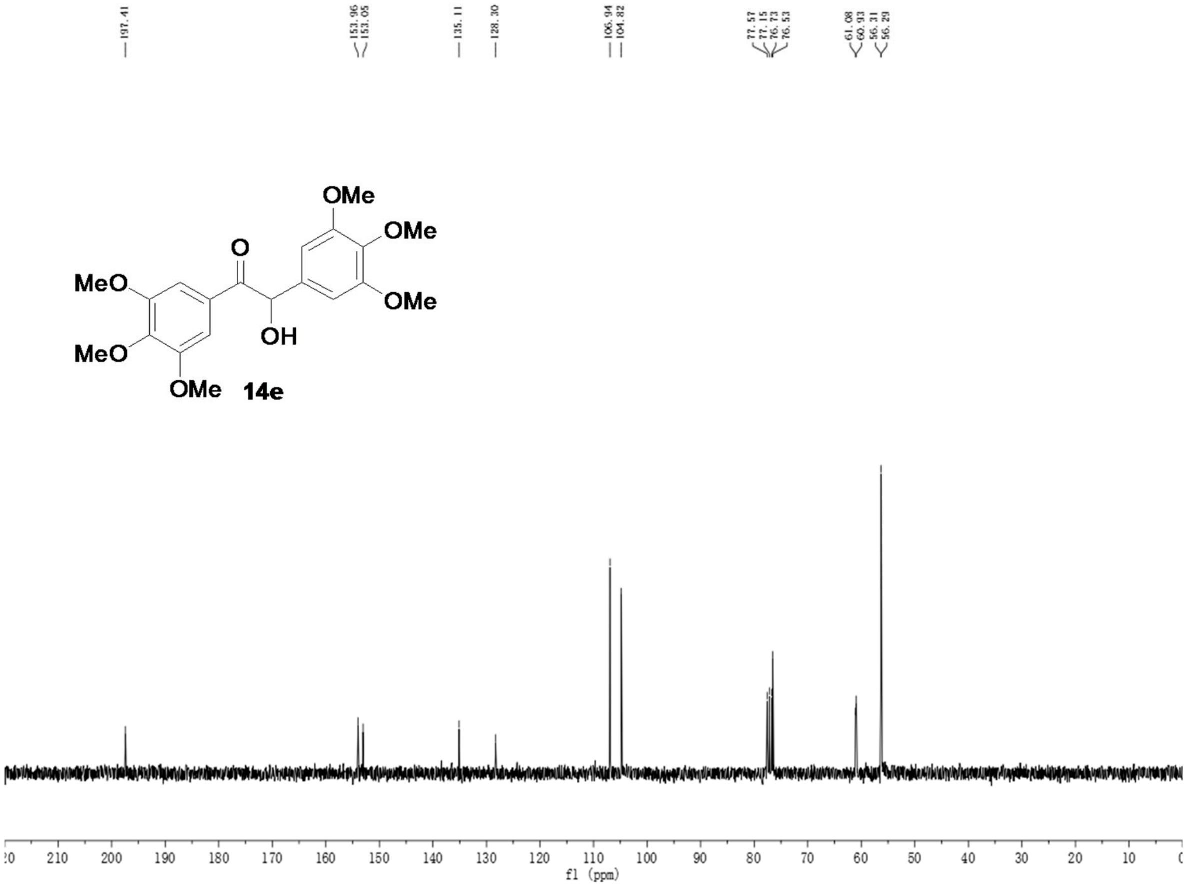

Figure S9. ^{1}H- and ^{13}C-NMR spectra for **1e**.

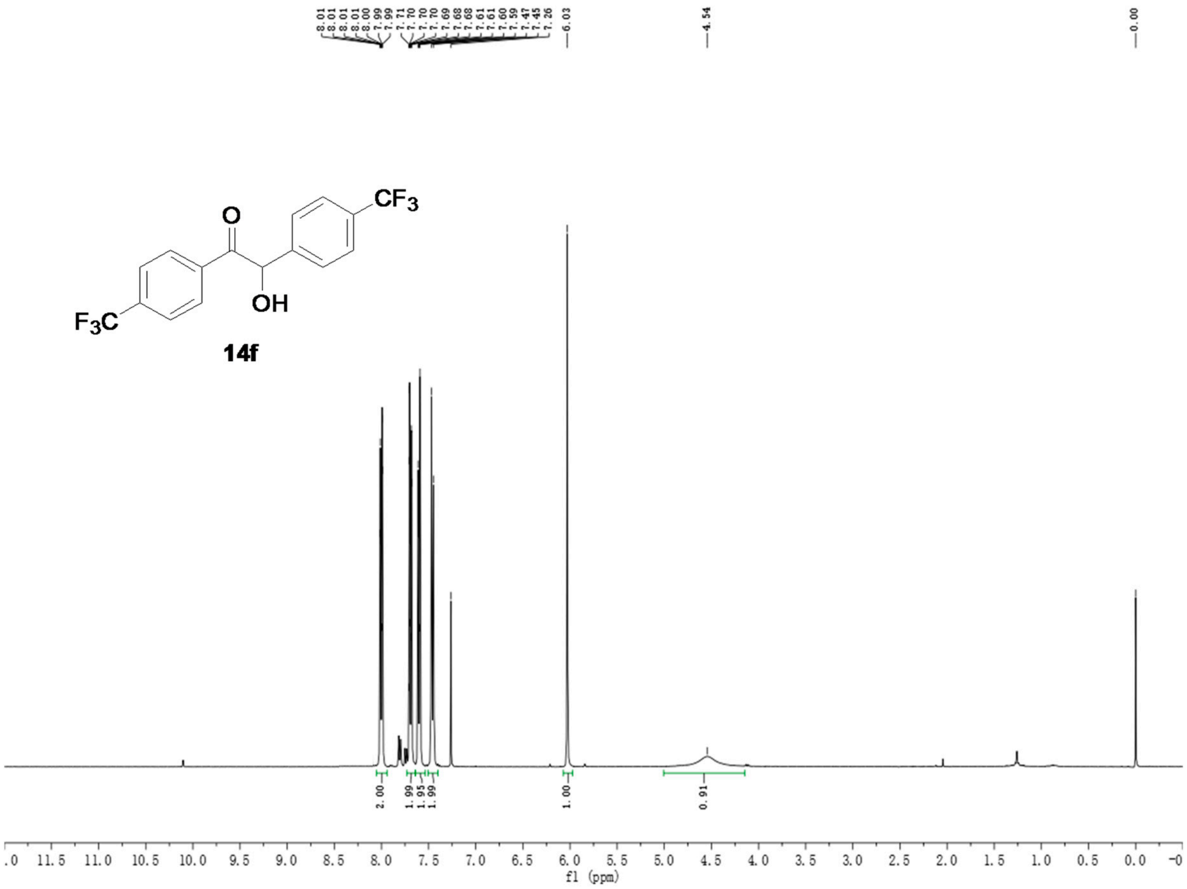

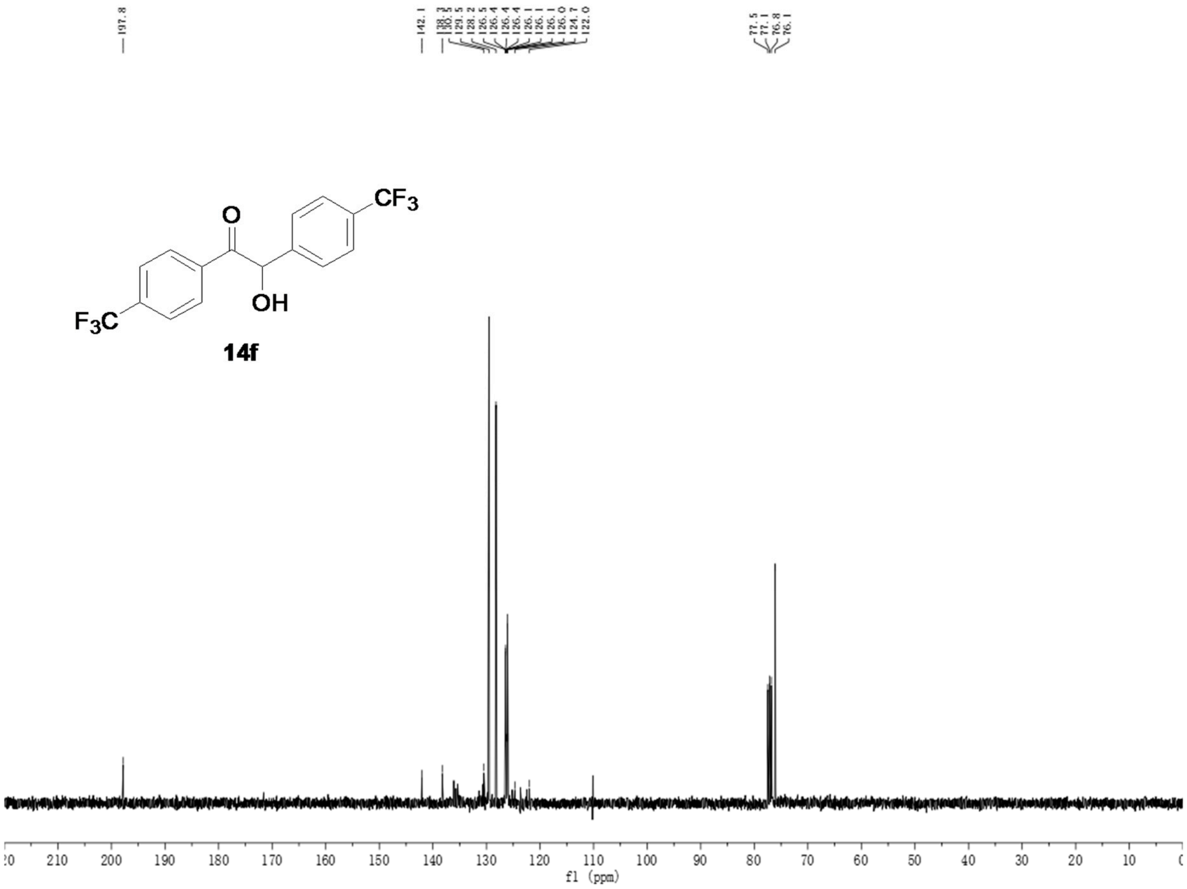

Figure S10. ^{1}H- and ^{13}C-NMR spectra for **14f**.

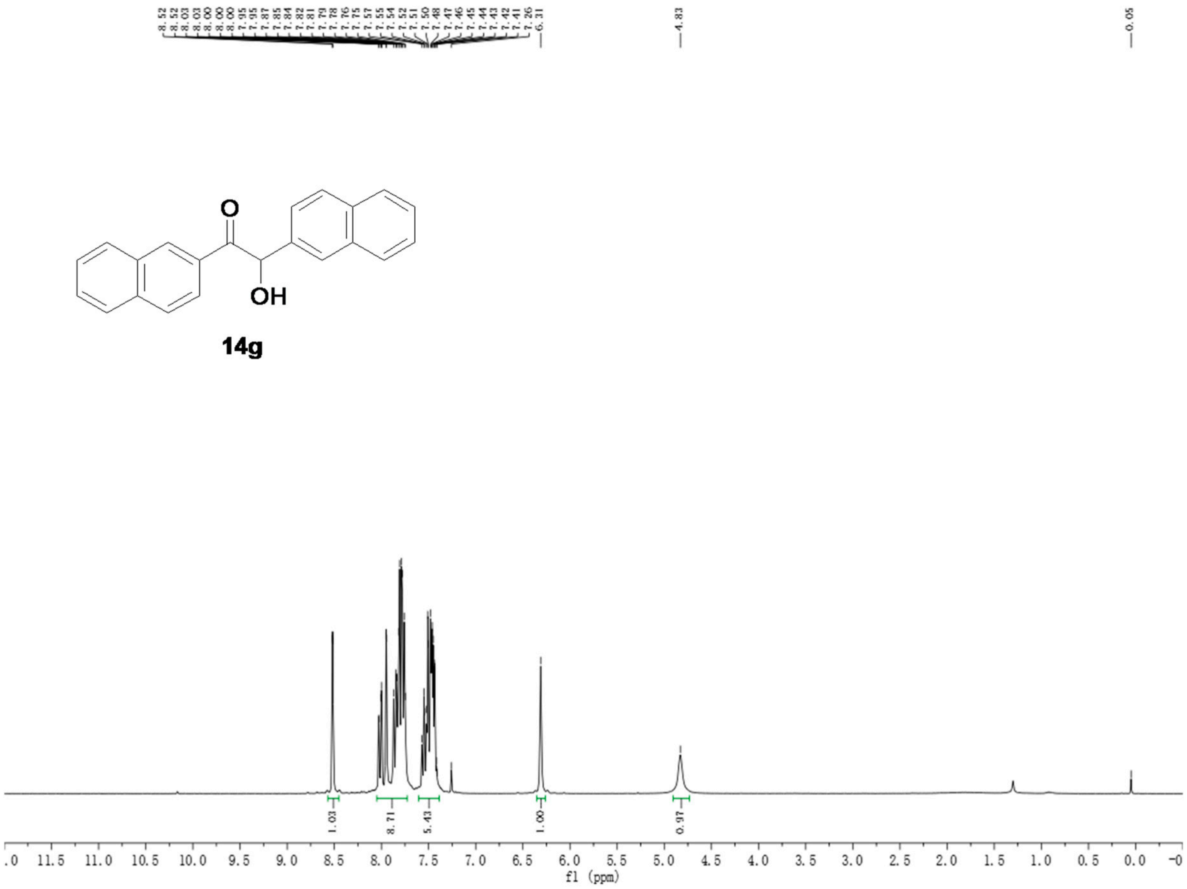

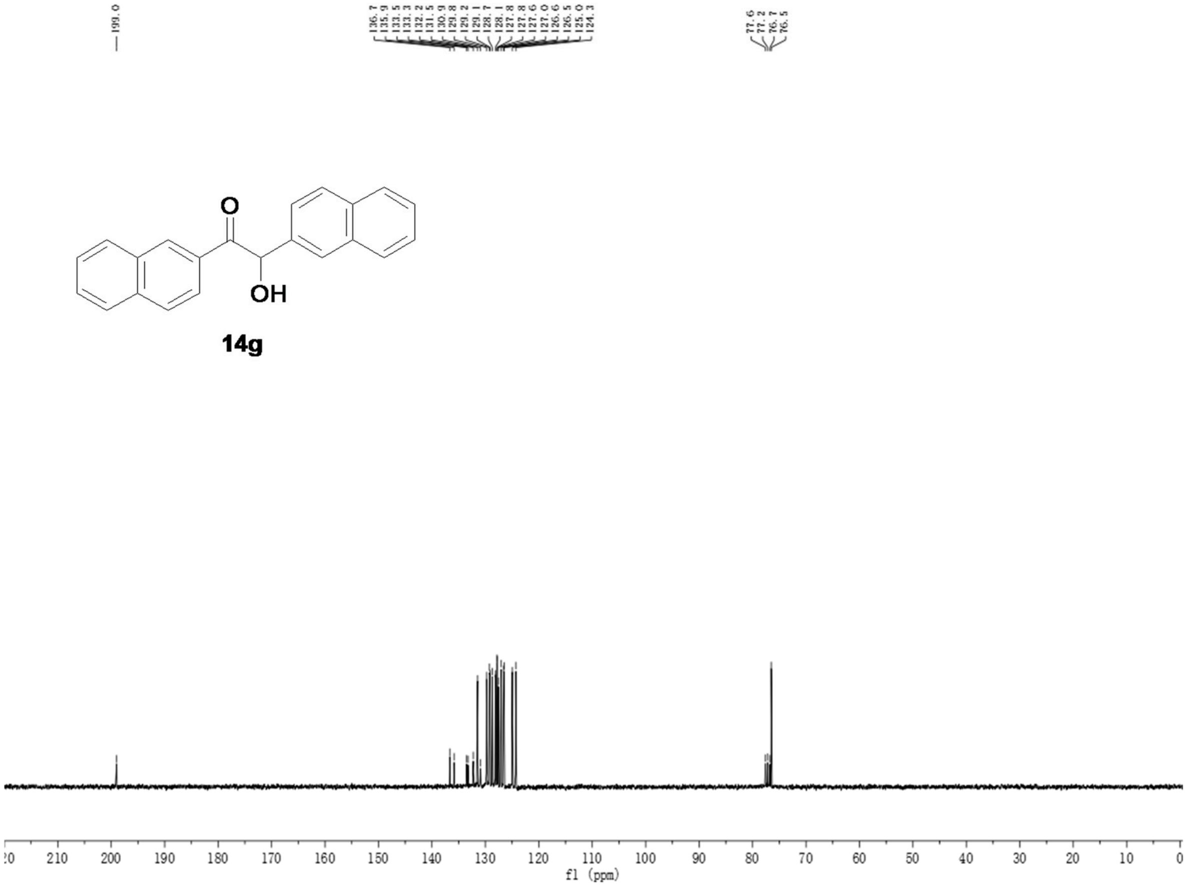

Figure S11. ^{1}H- and ^{13}C-NMR spectra for **14g**.

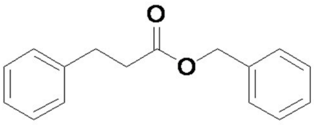

18Aa

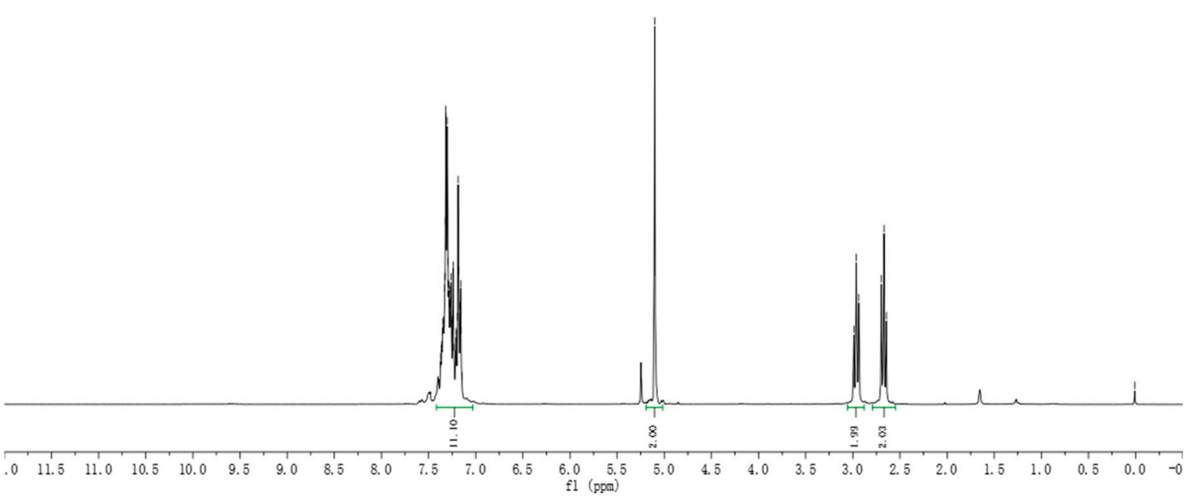

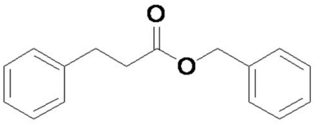

18Aa

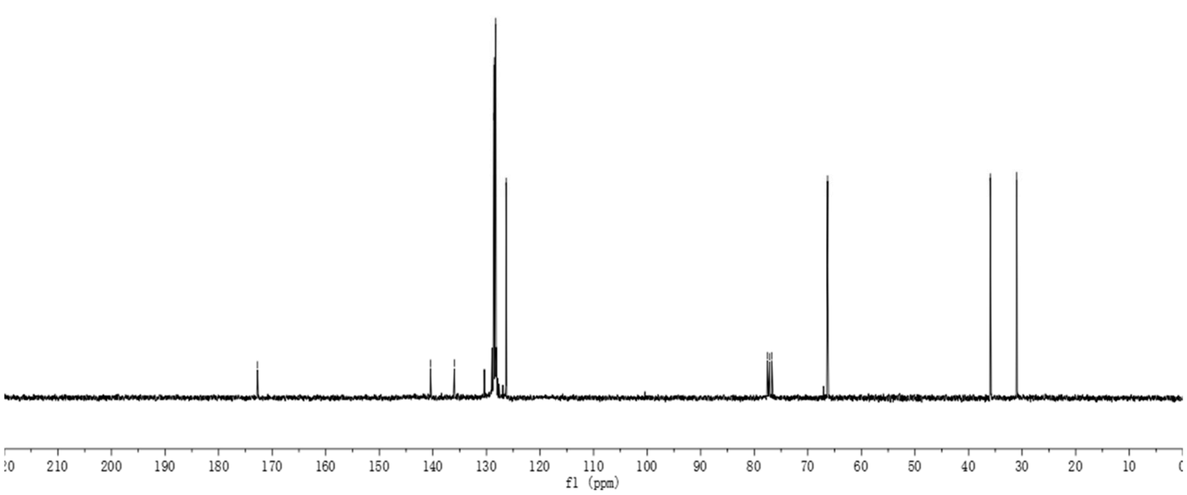

Figure S12. ^{1}H- and ^{13}C-NMR spectra for **18Aa**.

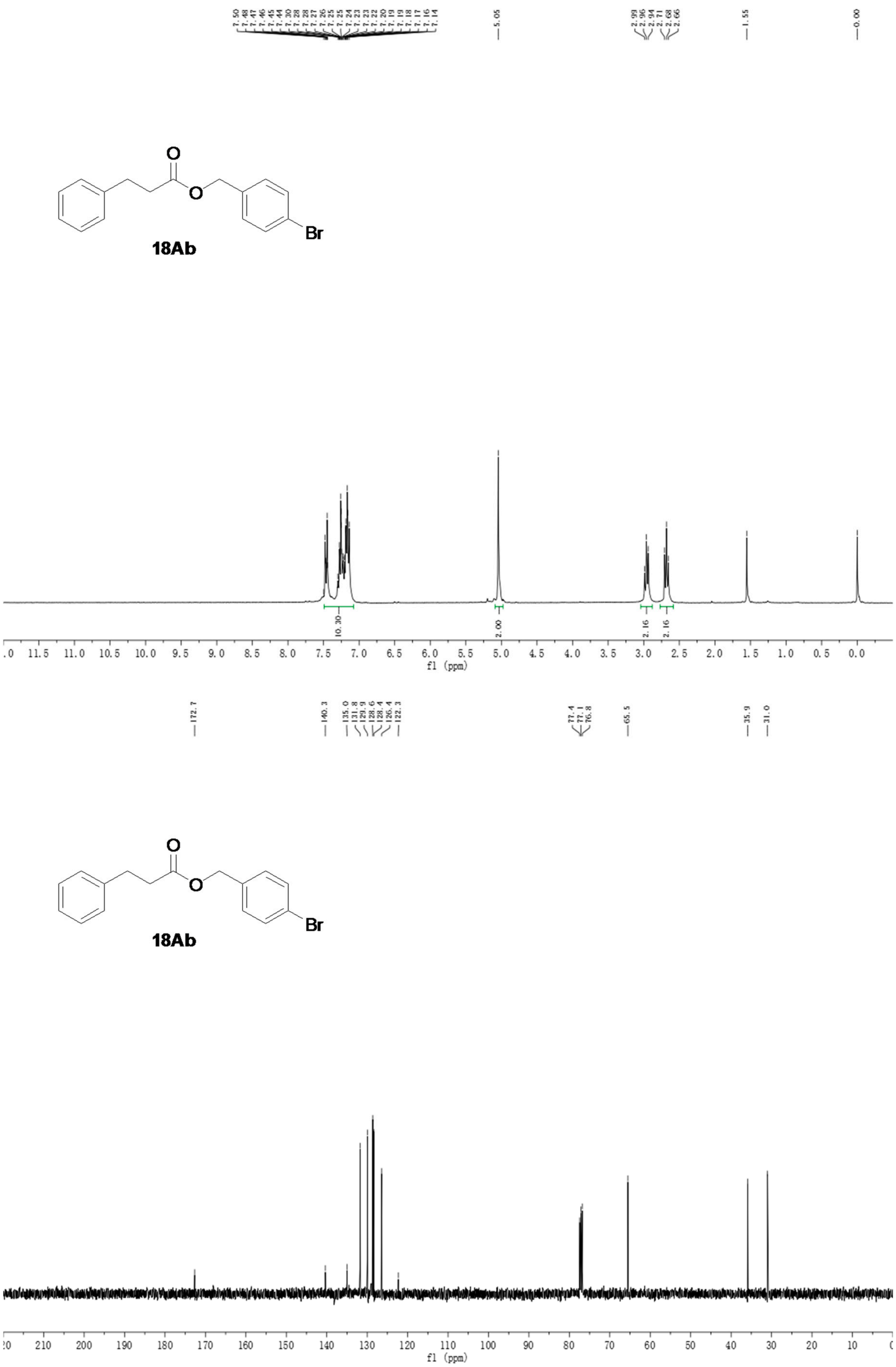

Figure S13. ^{1}H- and ^{13}C-NMR spectra for **18Ab**.

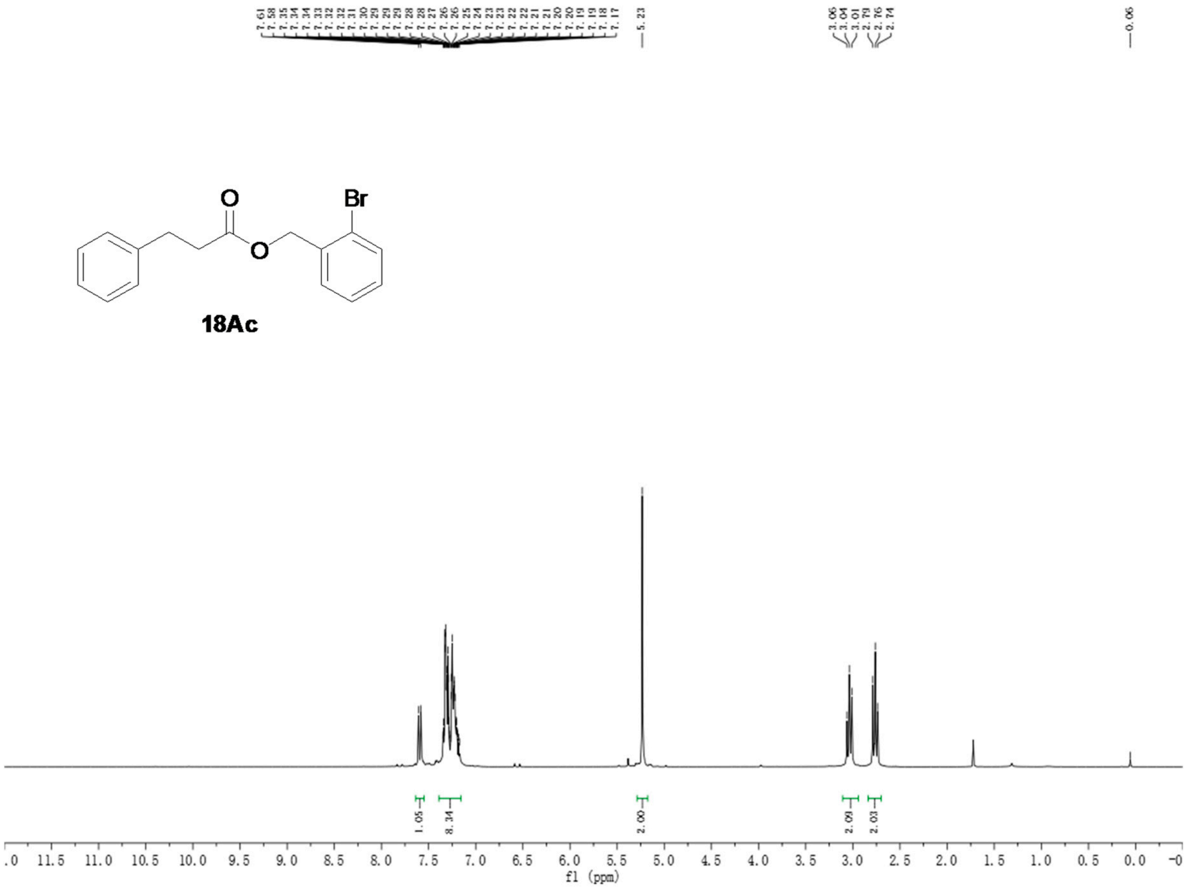

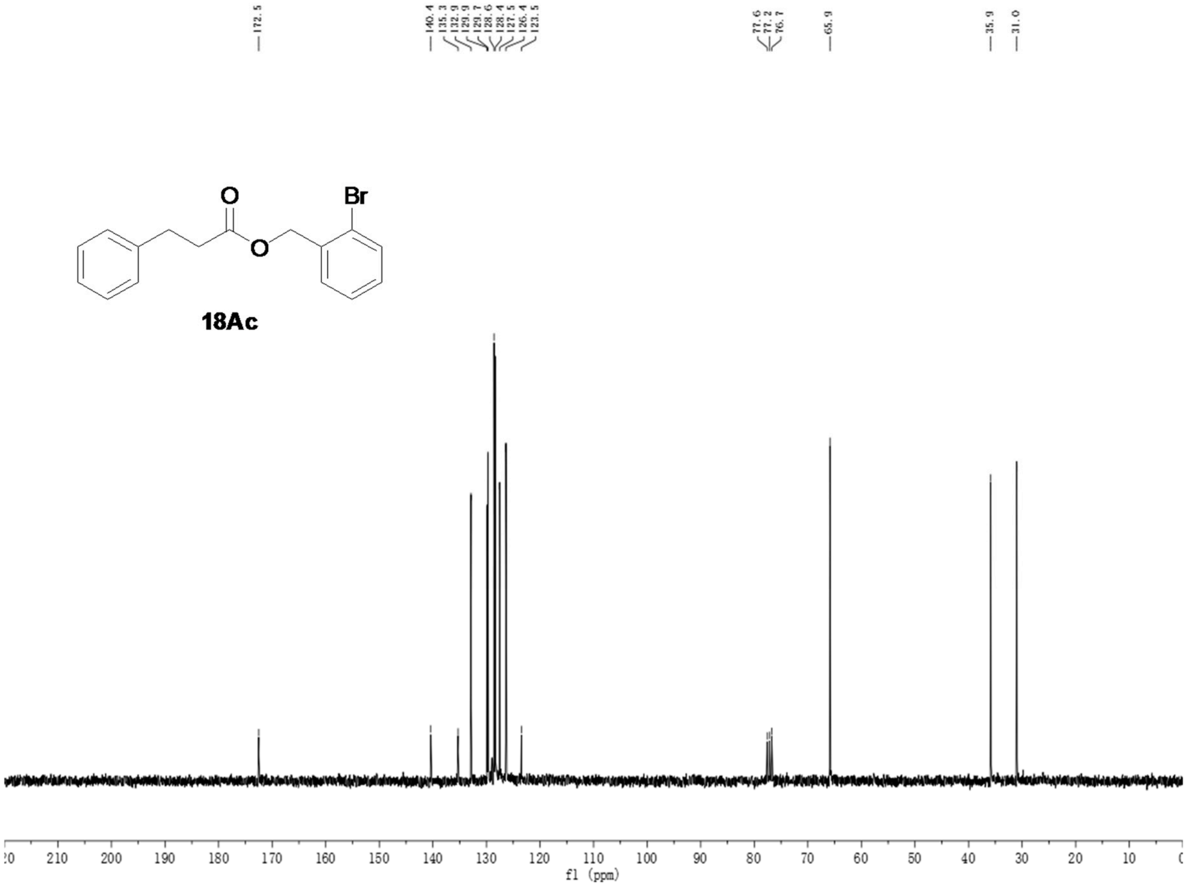

Figure S14. ^{1}H- and ^{13}C-NMR spectra for **18Ac**.

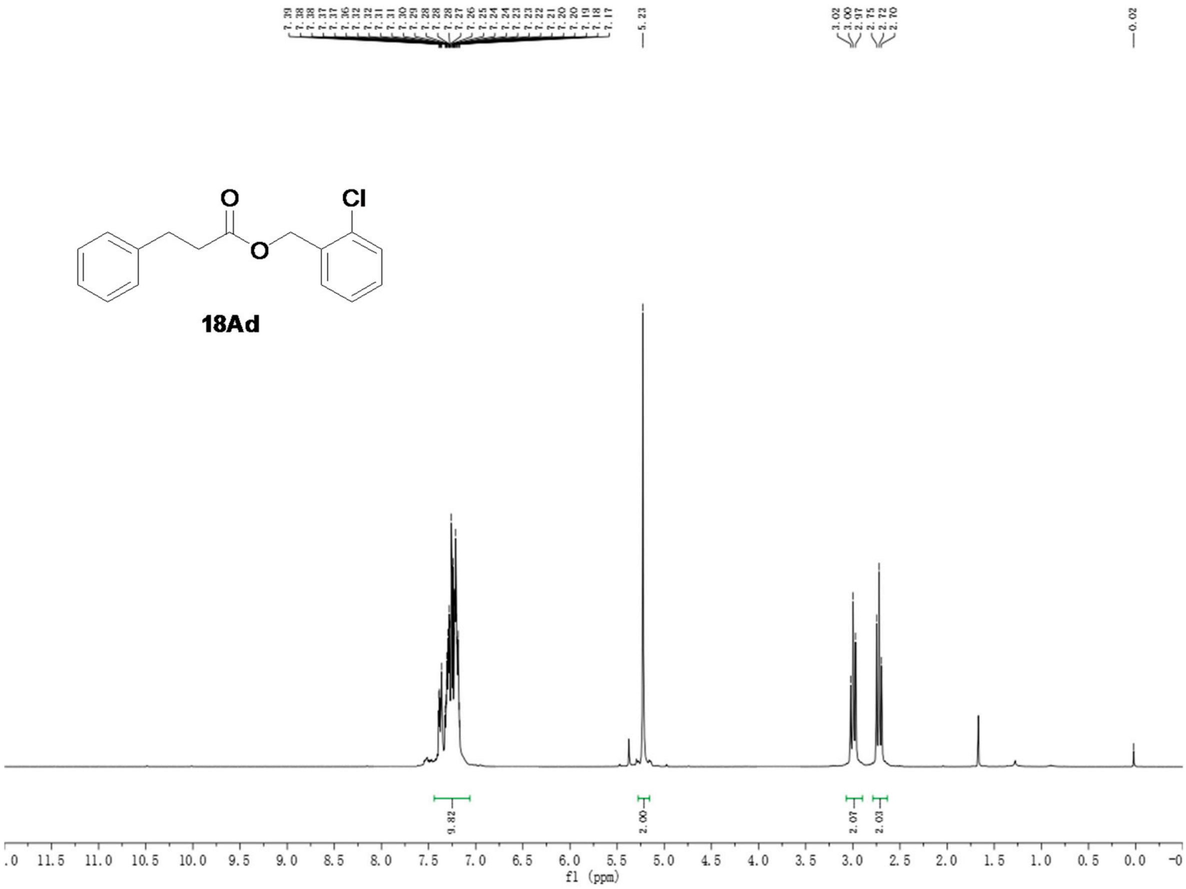

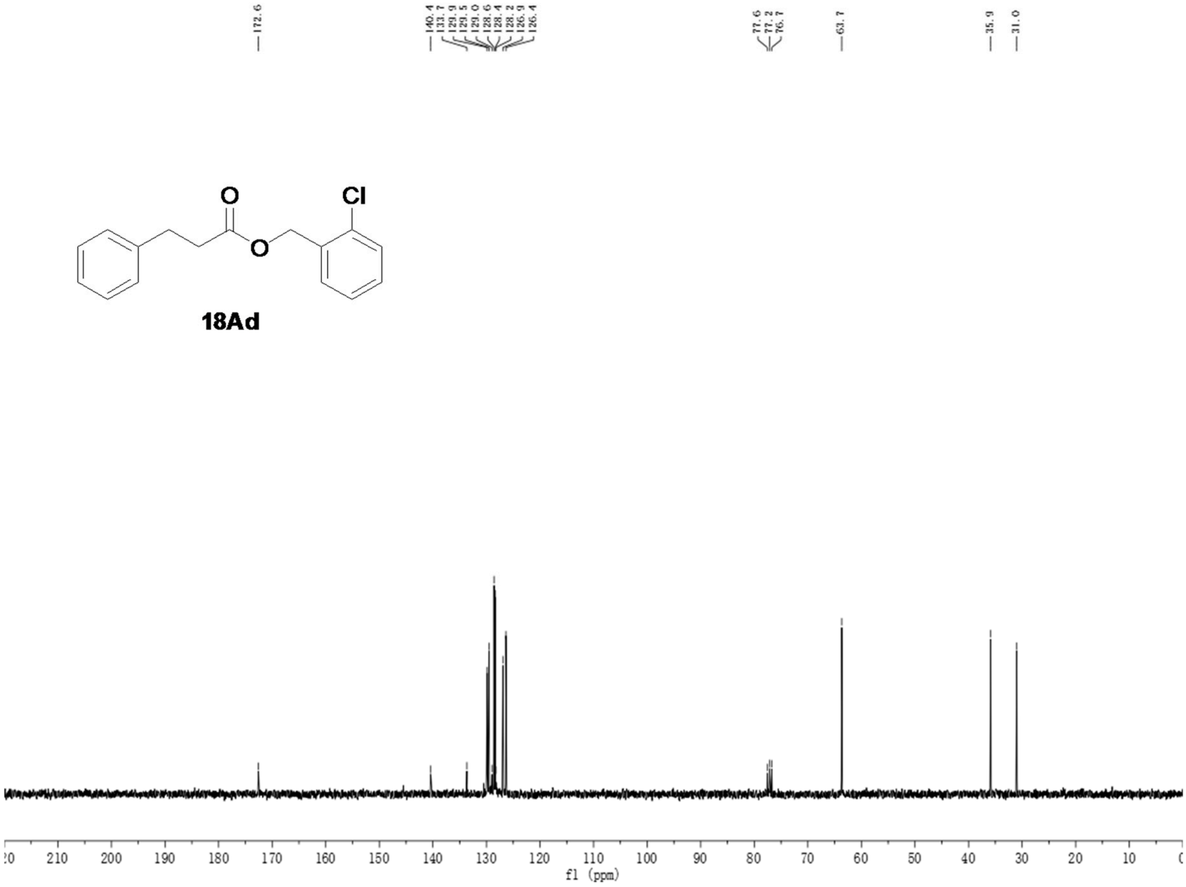

Figure S15. ^{1}H- and ^{13}C-NMR spectra for **18Ad**.

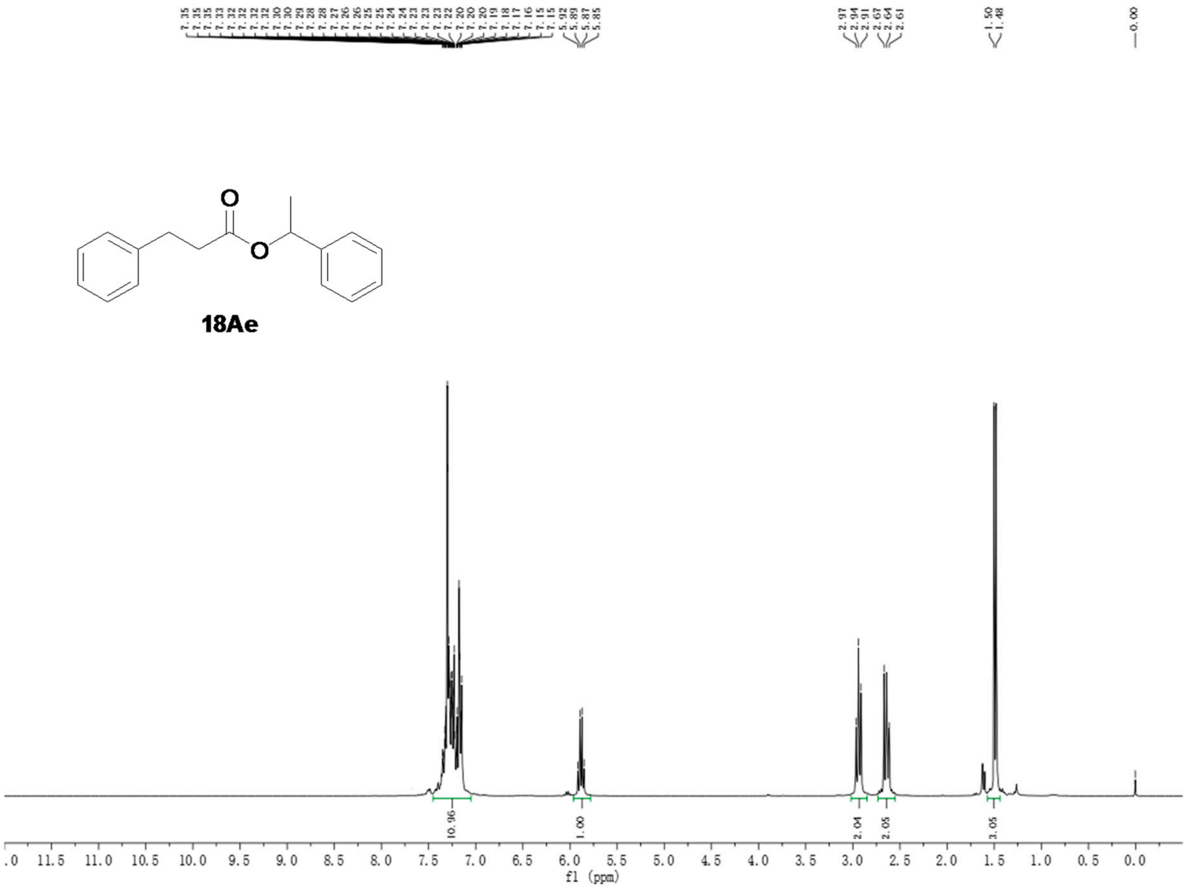

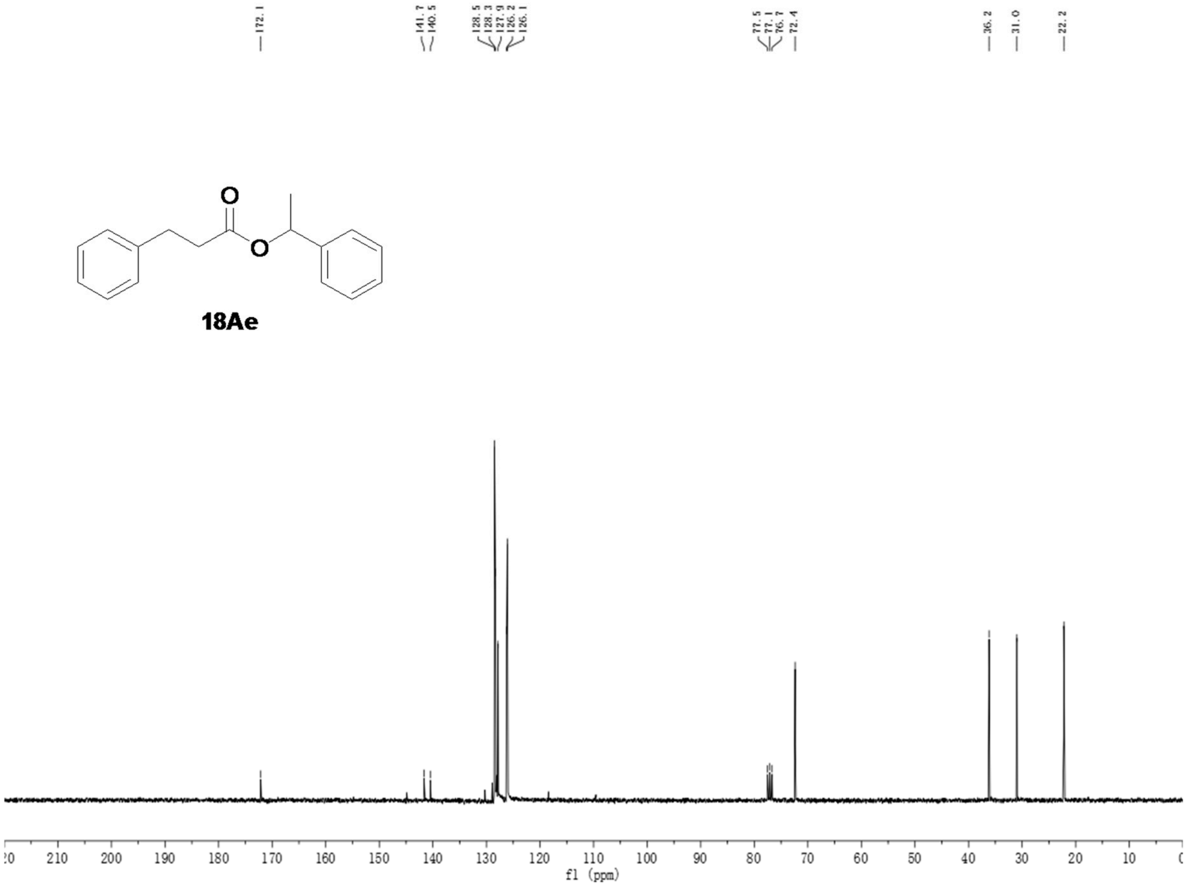

Figure S16. ^{1}H- and ^{13}C-NMR spectra for **18Ae**.

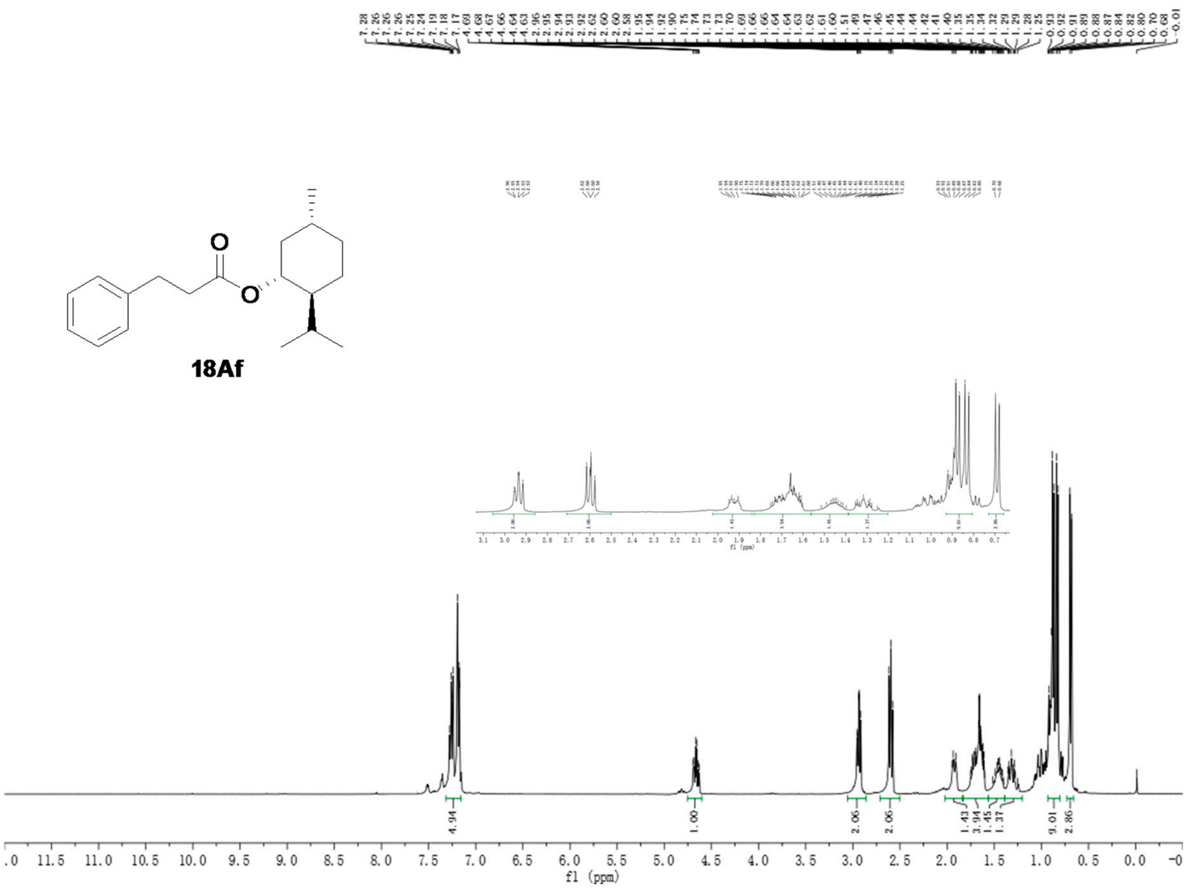

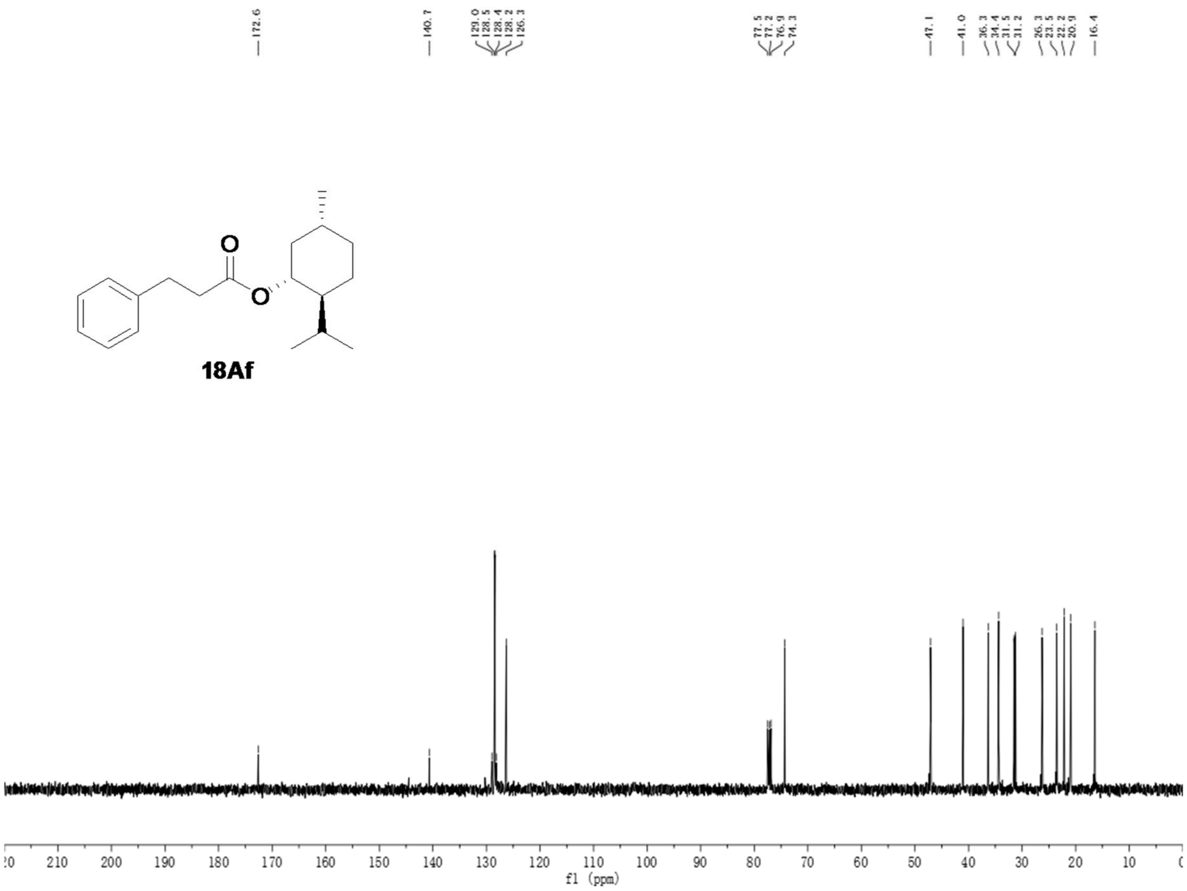

Figure S17. ^{1}H- and ^{13}C-NMR spectra for **18Af**.

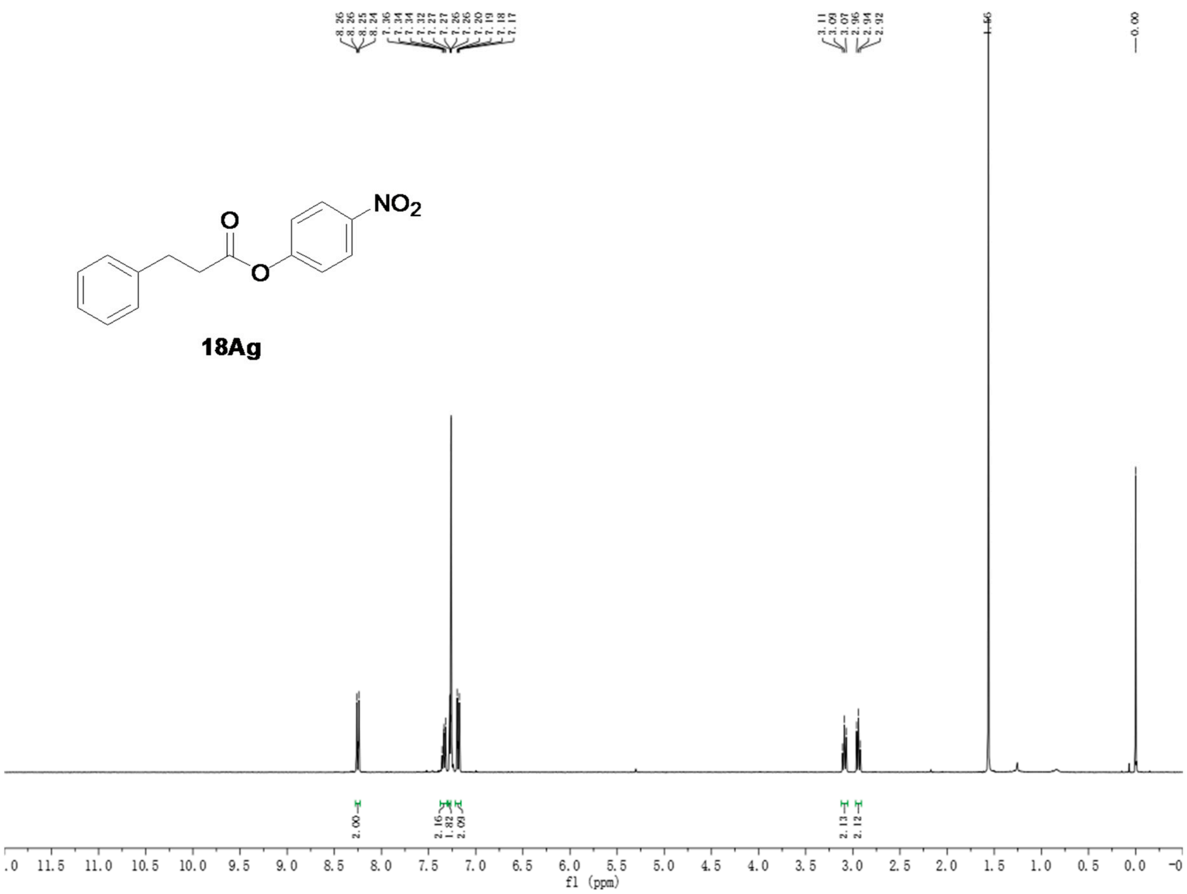

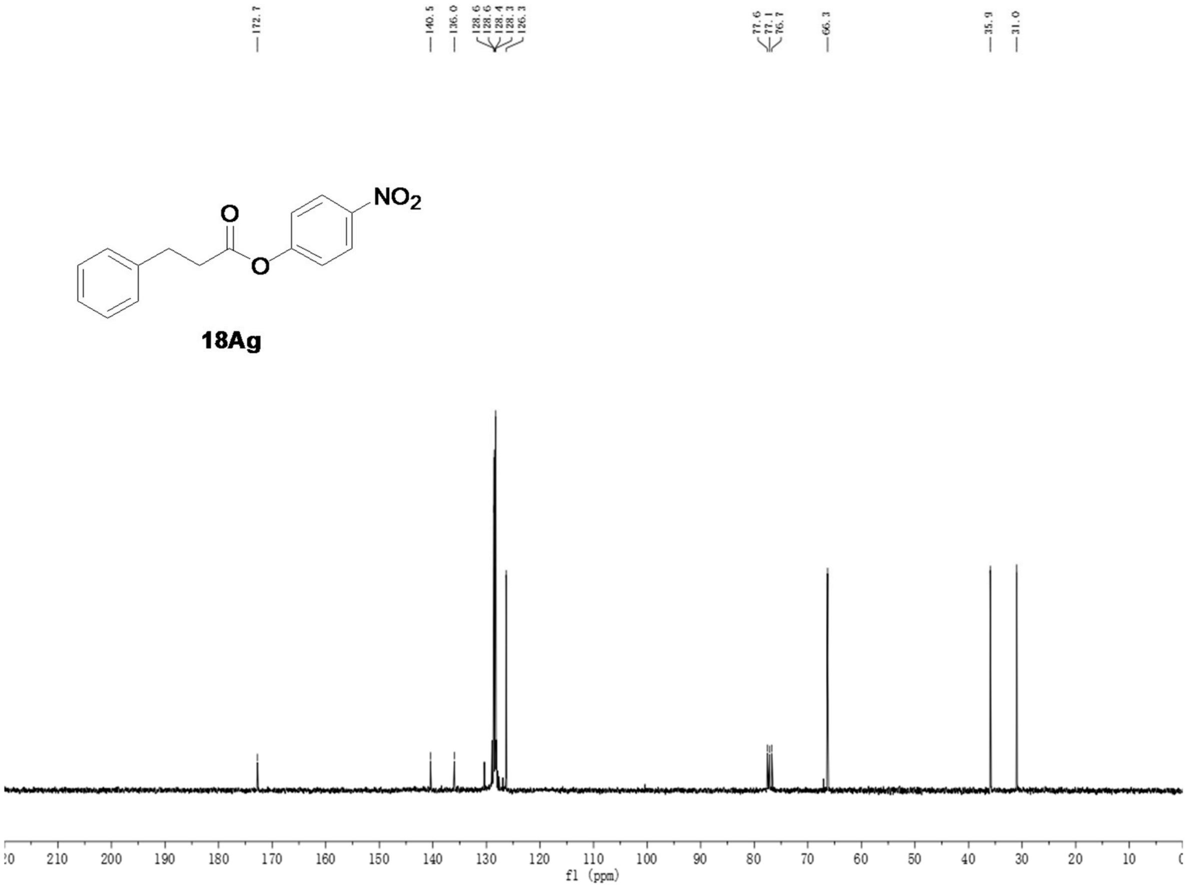

Figure S18. ^{1}H- and ^{13}C-NMR spectra for **18Ag**.

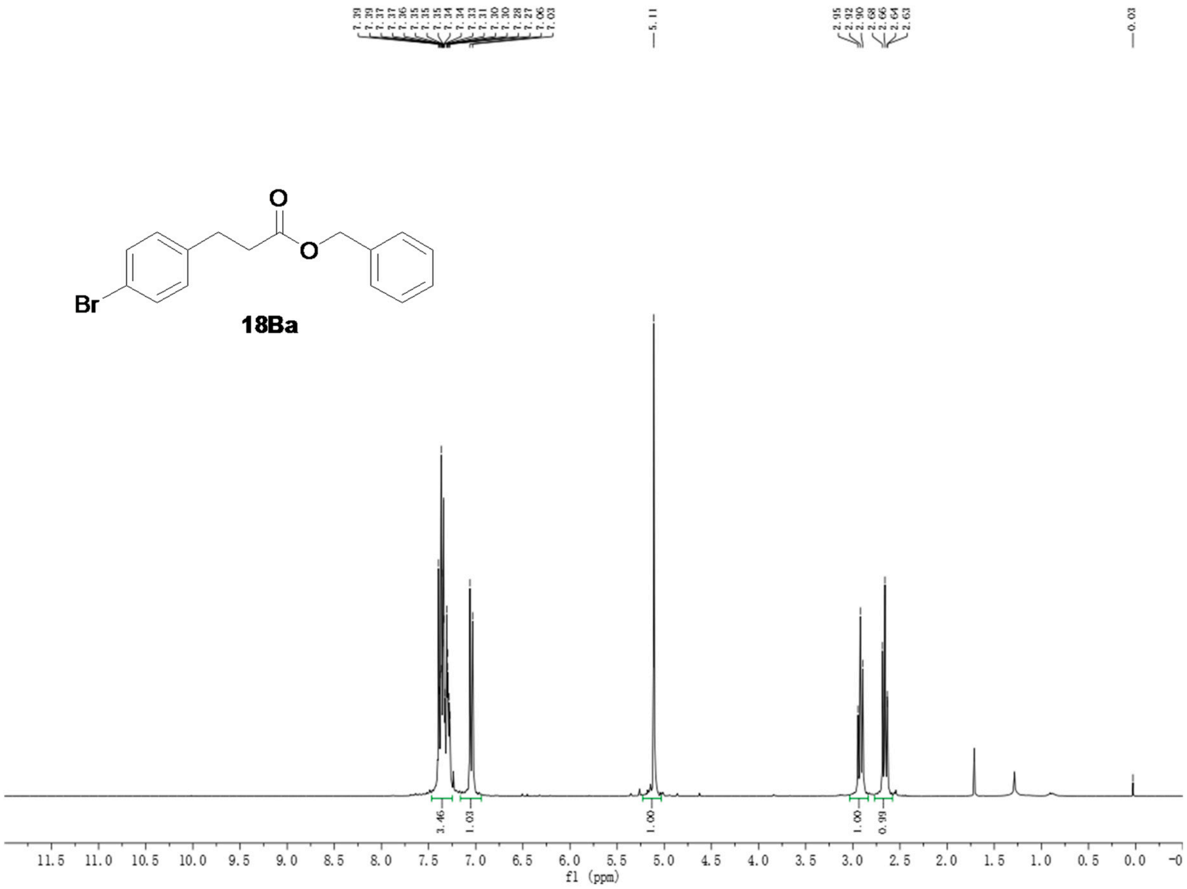

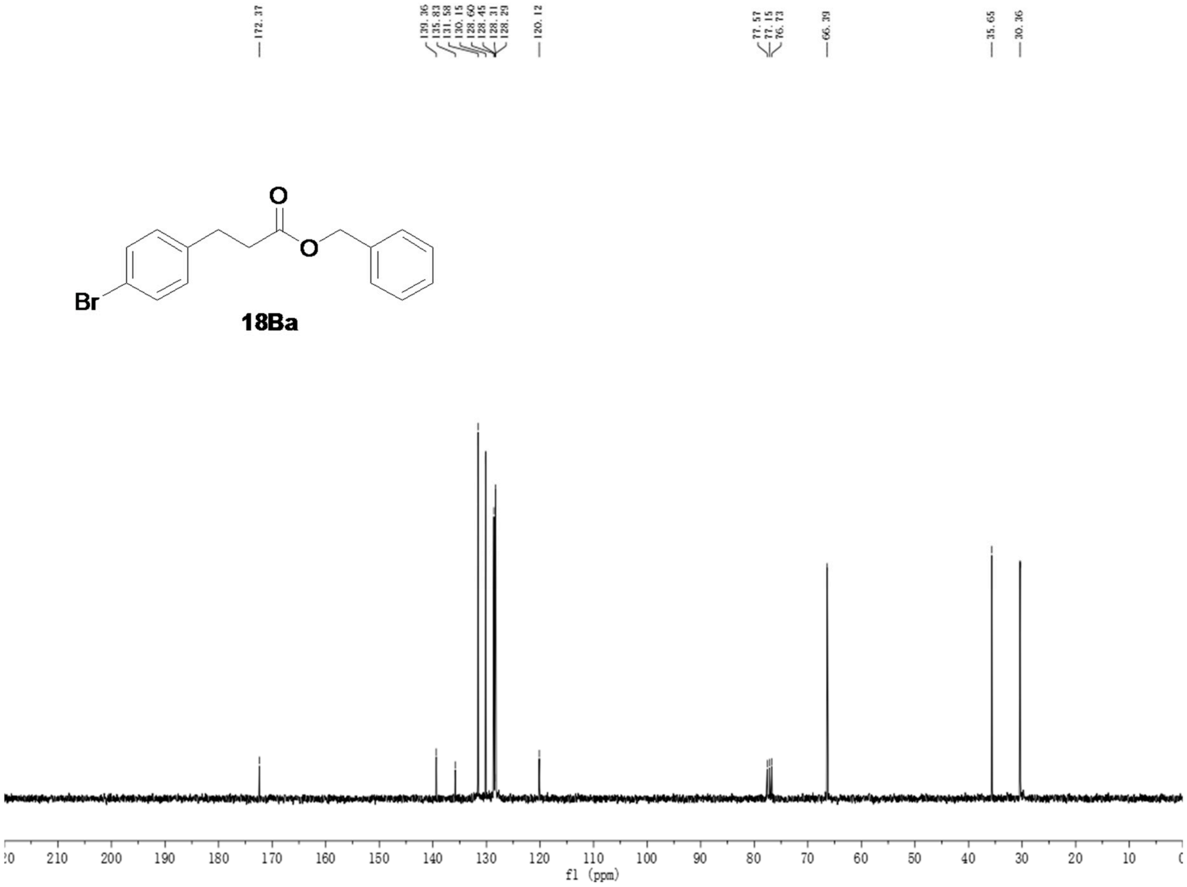

Figure S19. ^{1}H- and ^{13}C-NMR spectra for **18Ba**.

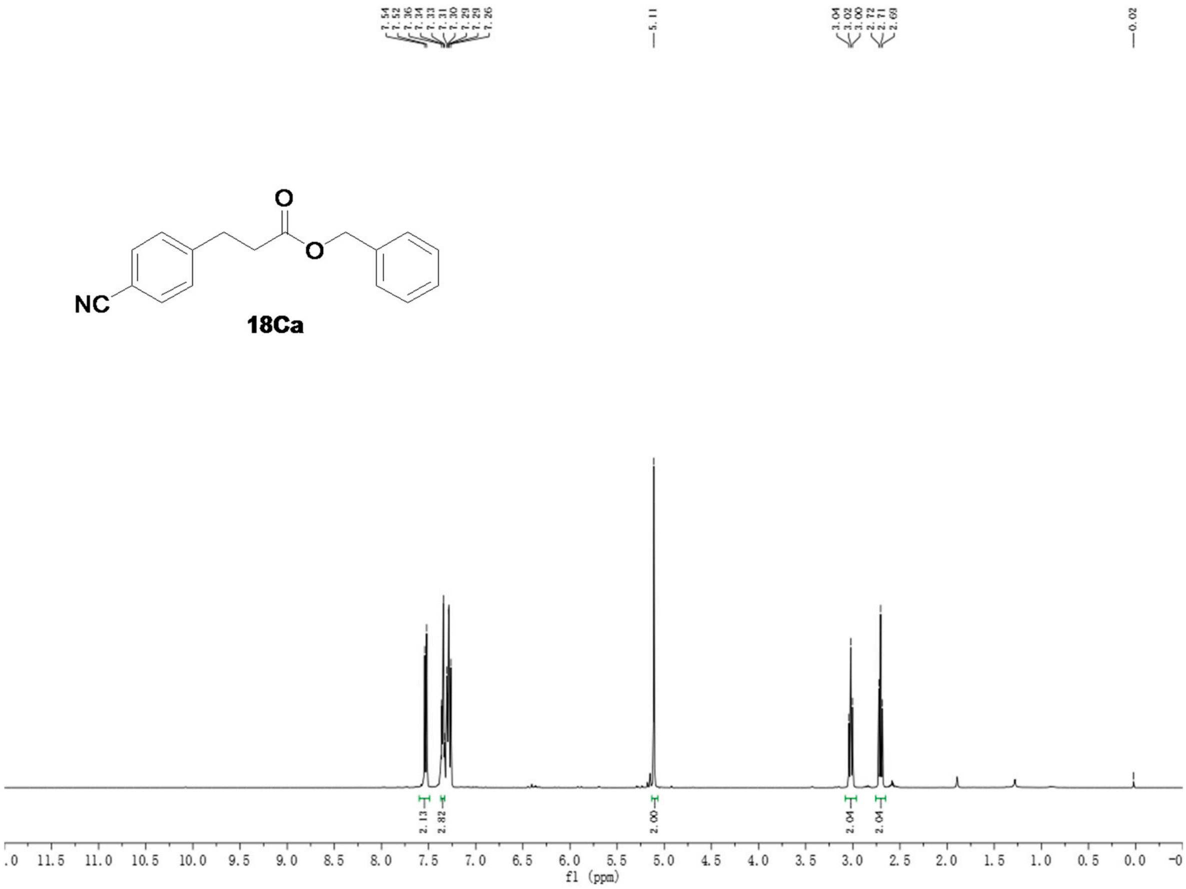

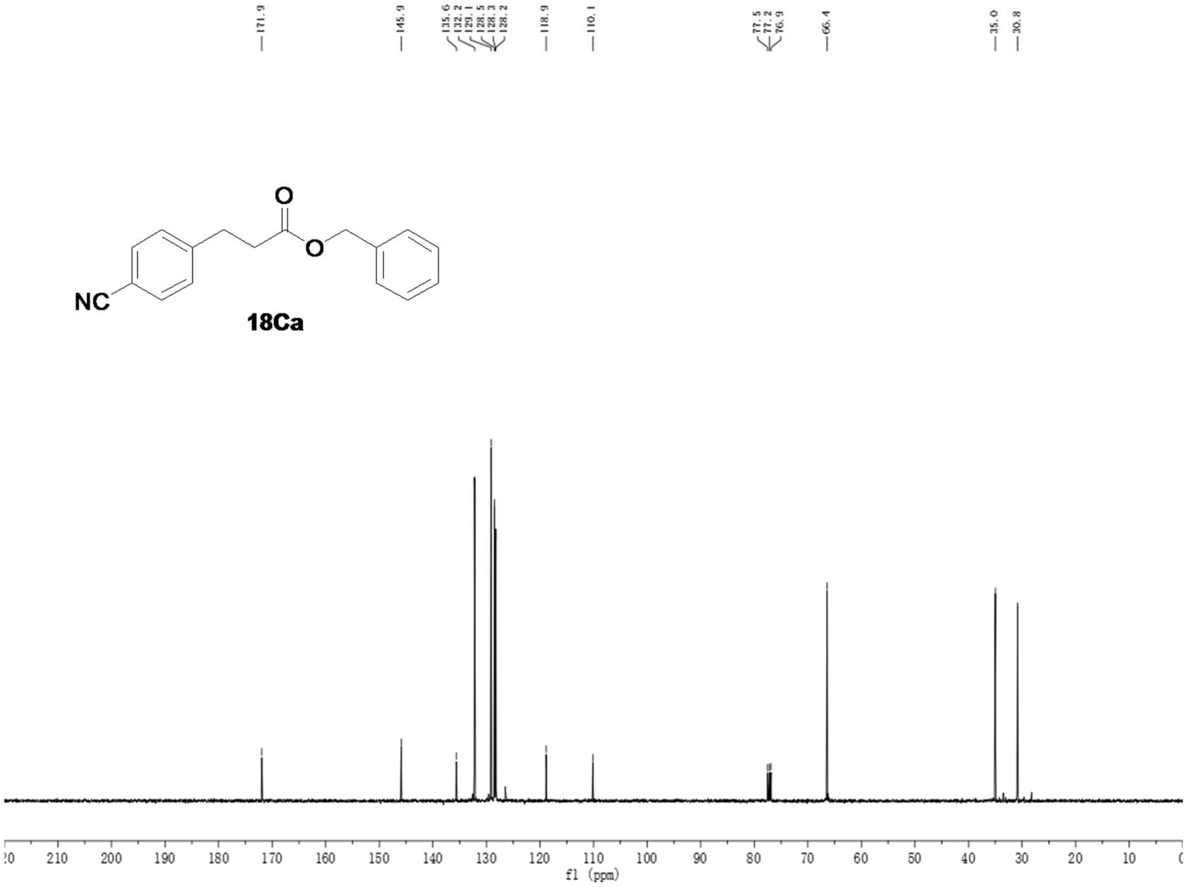

Figure S20. ^{1}H- and ^{13}C-NMR spectra for **18Ca**.

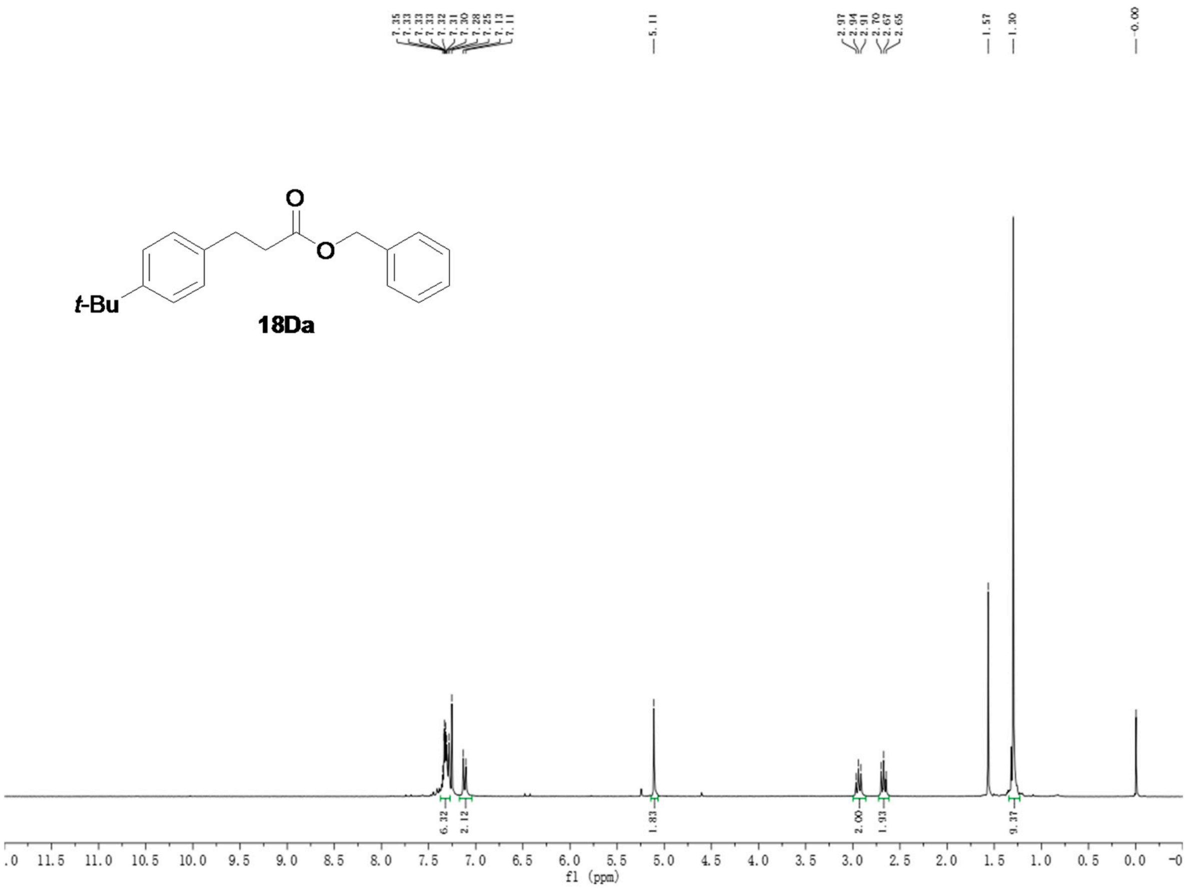

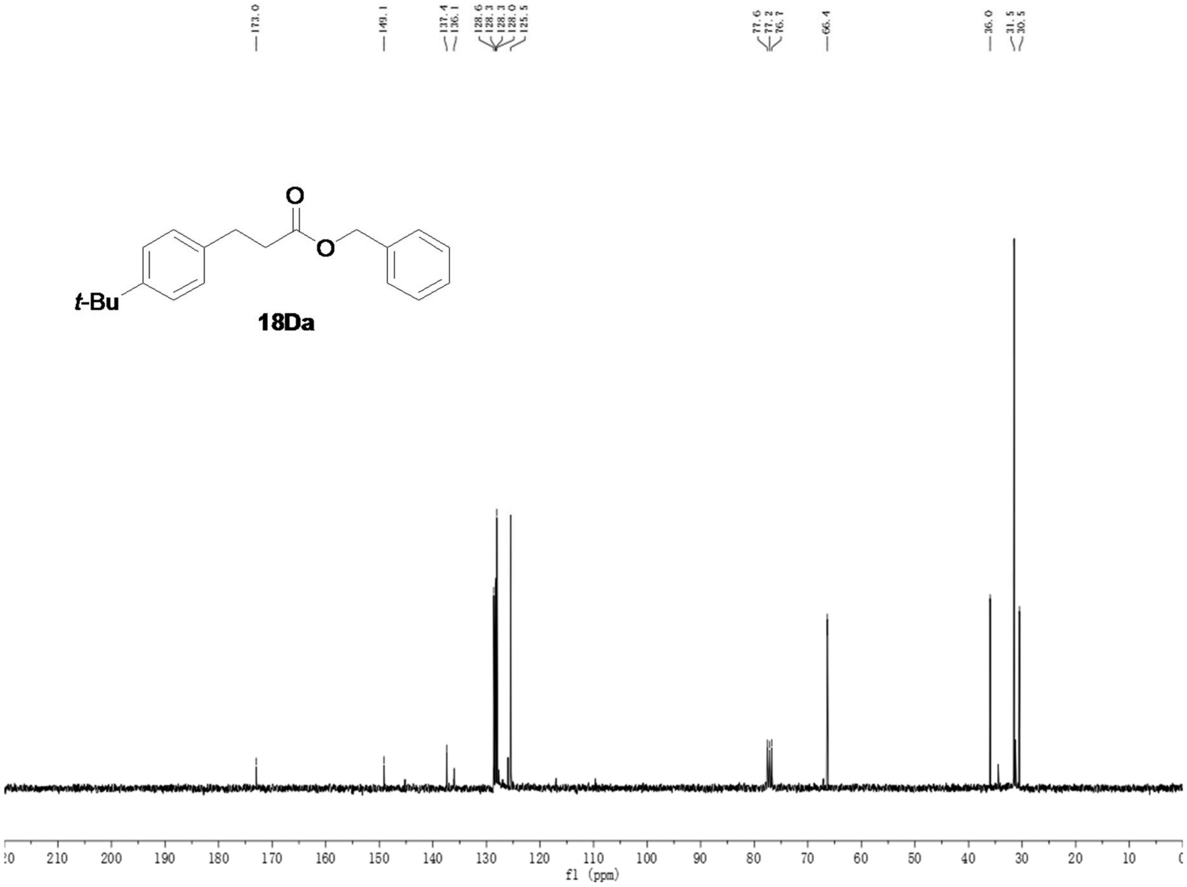

Figure S21. ^{1}H- and ^{13}C-NMR spectra for **18Da**.

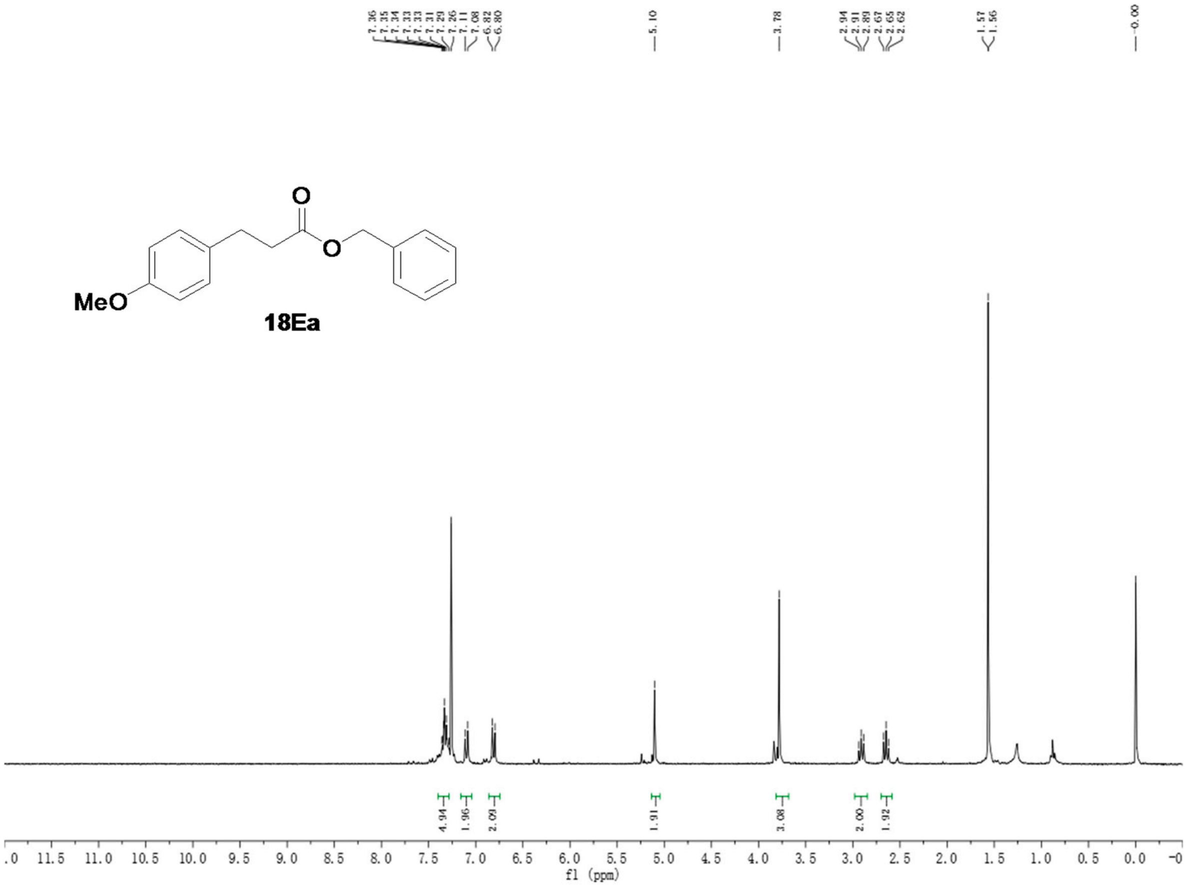

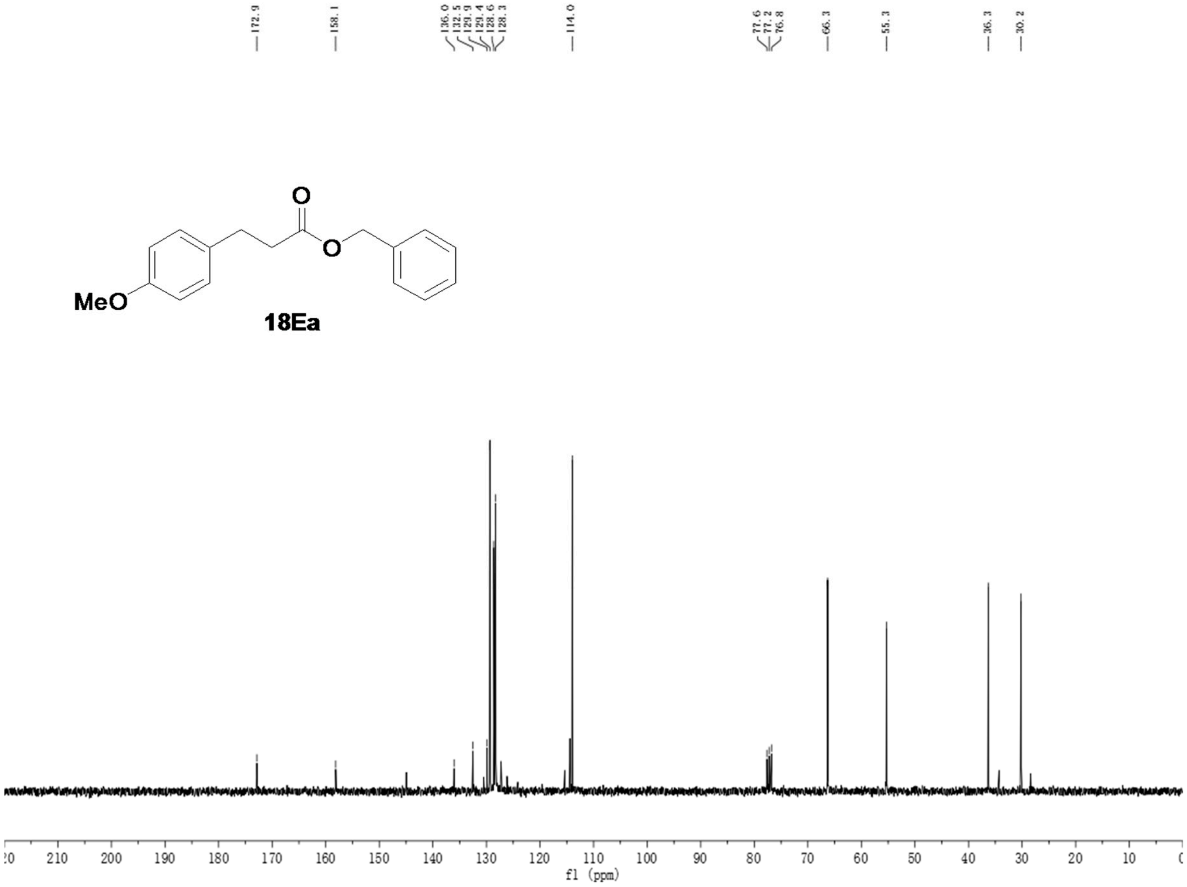

Figure S22. ^{1}H- and ^{13}C-NMR spectra for **18Ea**.

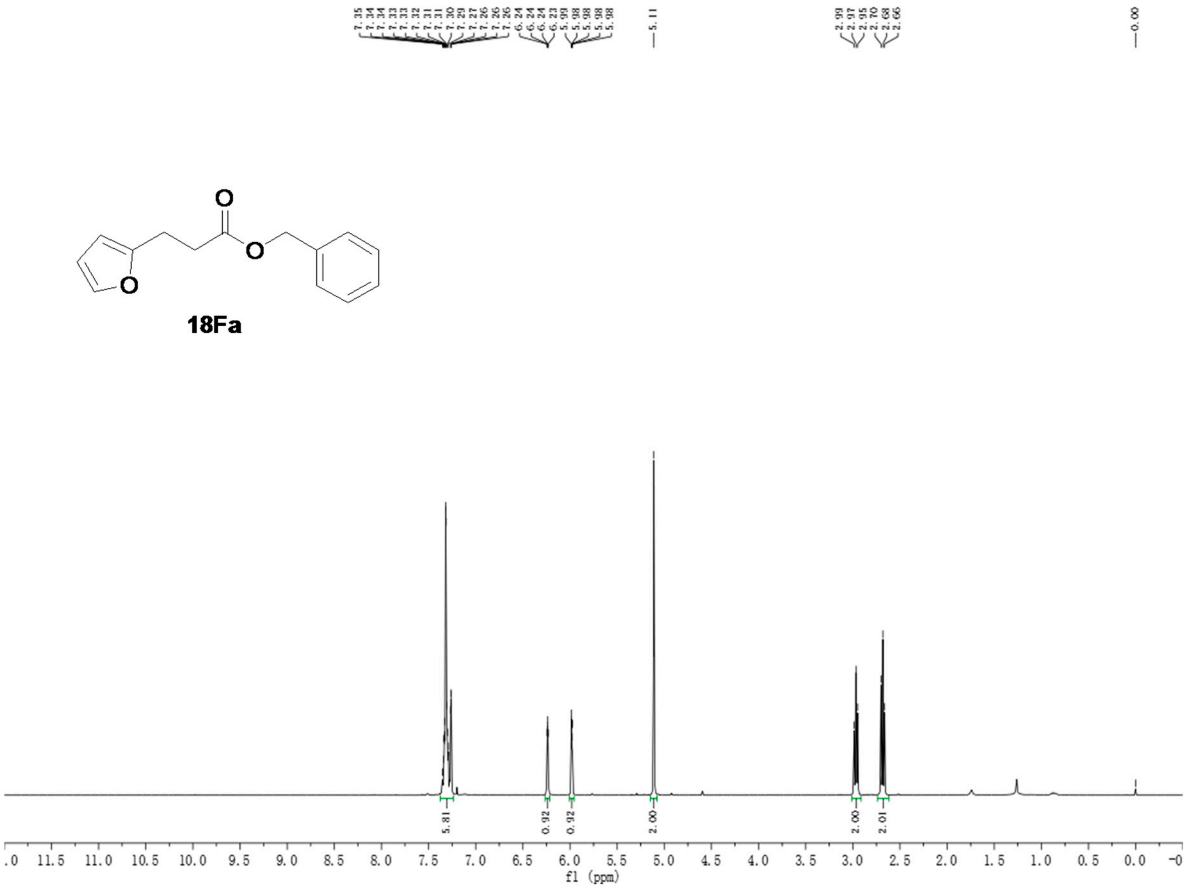

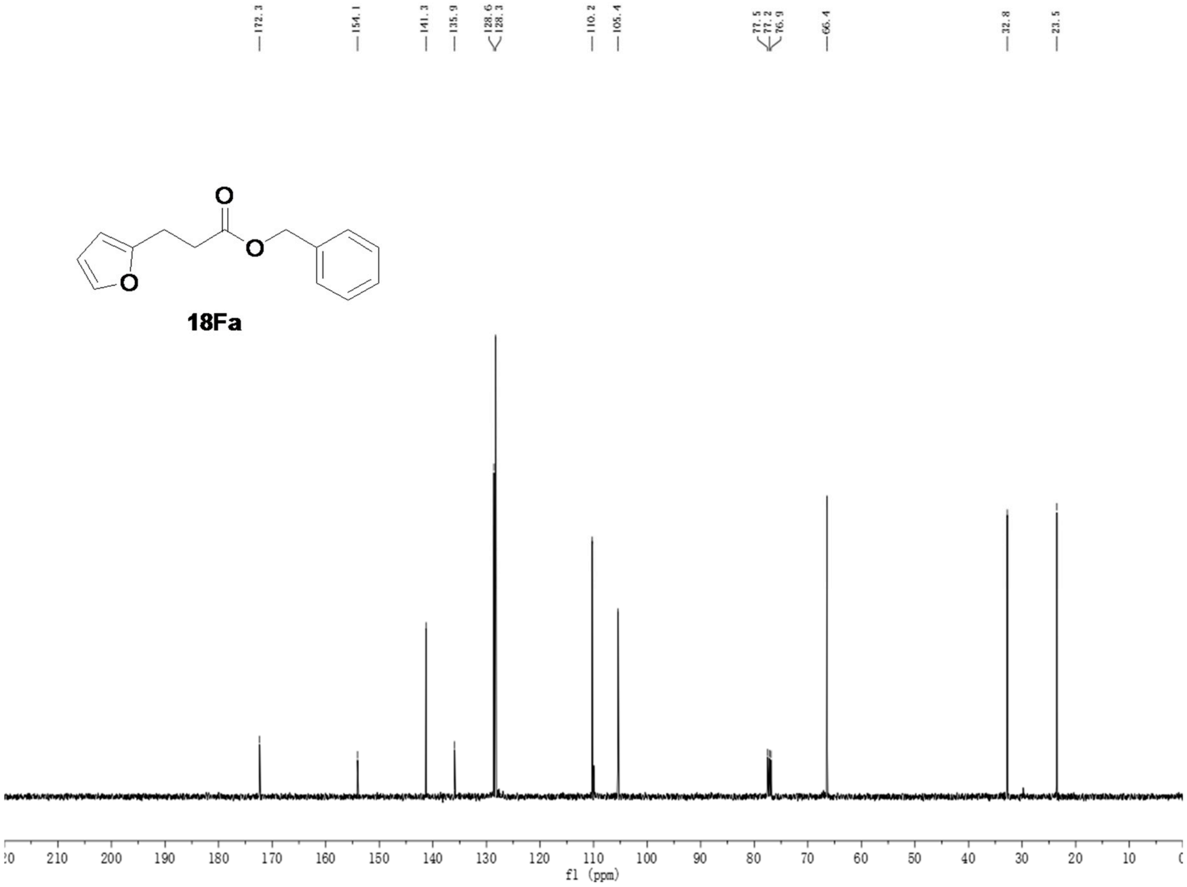

Figure S23. ^{1}H- and ^{13}C-NMR spectra for **18Fa**.
